# Natural products reshape osteosarcoma cell fate: promoting cell death

**DOI:** 10.3389/fphar.2026.1729552

**Published:** 2026-06-18

**Authors:** Binghan Yan, Junli Chang, Peng Zhao, Xingyuan Sun, Suxia Guo, Yanping Yang

**Affiliations:** 1 Longhua Hospital, Shanghai University of Traditional Chinese Medicine, Shanghai, China; 2 Key Laboratory of Theory and Therapy of Muscles and Bones, Ministry of Education, Shanghai, China

**Keywords:** cell fate, natural products, osteosarcoma, programmed cell death, therapeutic strategies

## Abstract

Osteosarcoma is the most prevalent primary malignant bone tumor in children and adolescents. Despite advancements in adjuvant chemotherapy and surgical techniques, the overall survival rate of osteosarcoma patients remains suboptimal, particularly in cases of metastatic or recurrent disease. Programmed cell death (PCD), a highly regulated process that includes apoptosis, autophagy, ferroptosis, pyroptosis, and necroptosis, plays a pivotal role in determining cellular fate (survival and death). In osteosarcoma, PCD dysregulation enables malignant cells to evade cell death signals, thereby accelerating tumor development. Natural products derived from various medicinal plants and dietary components exhibit promising potential for osteosarcoma treatment by regulating PCD through different mechanisms. With advantages such as low cost, minimal side effects, and wide availability, natural products have attracted considerable attention for translational research. This review systematically synthesizes current knowledge on PCD subtypes in osteosarcoma and elucidates the molecular mechanisms by which natural products regulate PCD to inhibit the development of osteosarcoma. By integrating these insights, we aim to offer novel perspectives for developing targeted therapeutic strategies and improving clinical outcomes in osteosarcoma patients.

## 1 Introduction

Osteosarcoma is the most common primary malignant bone tumor, characterized by aggressive local invasion, frequent disease recurrence, and the early development of distant metastasis. The lesions are typically located at the ends of long bones, such as the femur, tibia, and humerus, and are prone to metastasize to the lungs (Bhattasali et al., 2015). Osteosarcoma originates from mesenchymal stem cells with osteogenic potential and is characterized by tumor cells proliferating to form immature bones or bone-like tissues. An epidemiological survey of osteosarcoma indicates a bimodal age distribution, with a higher incidence in both adolescents and individuals over 60 years old, and a slightly higher prevalence among males than females. Osteosarcoma is one of the most common malignant tumors among children, making it a leading cause of cancer-related deaths in this population (Kansara et al., 2014). The standard treatment for osteosarcoma is a neoadjuvant chemotherapy-surgery-consolidation chemotherapy regimen, which has improved the survival rate of patients (Anderson, 2016). However, the 5-year survival rate remains unsatisfactory, especially for patients with recurrent or metastatic disease.

One of the major obstacles to effective osteosarcoma treatment is the development of drug resistance, which is closely linked to the evasion of cell death mechanisms. Cell death can be classified into non-programmed cell death and PCD, with PCD gaining significant attention due to its precise regulatory role in maintaining homeostasis and responding to environmental changes (Li et al., 2023a). In recent years, studies have shown that tumor cells can escape treatment pressure and form a drug-resistant phenotype by abnormally regulating PCD pathways. For instance, traditional chemotherapeutic agents such as cisplatin and doxorubicin rely on the activation of apoptotic pathways, particularly the mitochondrial caspase cascade. However, osteosarcoma cells often exhibit downregulation of pro-apoptotic proteins and overexpression of anti-apoptotic proteins, elevating the threshold for apoptosis initiation (Gonçalves et al., 2015; Pistritto et al., 2016). Additionally, when apoptosis is suppressed, necroptosis mediated by receptor-interacting protein kinase-3 (RIPK3)/Mixed Lineage Kinase domain-Like (MLKL) can serve as an alternative cell death mechanism (Karlowitz and van Wijk, 2023). However, osteosarcoma cells may downregulate RIPK3 or impair MLKL phosphorylation, thereby blocking necroptosis and resisting tumor necrosis factor (TNF)-α-based therapies (Morgan and Kim, 2022; Sharma et al., 2025). Furthermore, autophagy, as an adaptive cell survival mechanism, plays a critical role in chemotherapy resistance in osteosarcoma. Studies have shown that Sestrin2 activates the PERK-eIF2α-CHOP signaling pathway to inhibit mTOR, thereby inducing protective autophagy. This process is also accompanied by the downregulation of apoptosis-related regulators such as Bcl-2, ultimately enhancing osteosarcoma cell resistance to chemotherapeutic agents. Together, these mechanisms constitute a network of PCD evasion, representing one of the major causes of therapeutic failure in osteosarcoma (Tang Z. et al., 2021). Therefore, PCD dysregulation plays a crucial role in the progression and drug resistance of osteosarcoma (Liu S. et al., 2024). Therefore, targeting specific regulatory molecules to modulate PCD represents a promising therapeutic approach for inhibiting osteosarcoma progression. Studies have shown that natural products derived from various fruits, vegetables, and medicinal plants can regulate PCD through different mechanisms, demonstrating great potential in the prevention and treatment of osteosarcoma. These natural compounds are increasingly regarded as promising complementary and alternative therapeutic agents due to their cost-effectiveness and reduced adverse effects compared to conventional treatments.

Unlike previous reviews that usually focus on one or two PCD subtypes or a single mechanistic pathway, this work aims to review several common forms of PCD in osteosarcoma, including apoptosis, autophagy, ferroptosis, pyroptosis, and necroptosis, and to explore their potential links to osteosarcoma development. Furthermore, we introduce and contextualize the emerging concept of PANoptosis. Moreover, we also include the roles and mechanisms of natural products in regulating PCD in osteosarcoma treatment, thereby providing novel insights and theoretical support for developing therapeutic strategies against osteosarcoma.

## 2 Natural products and apoptosis in osteosarcoma

### 2.1 Overview of apoptosis

In 1972, Kerr and colleagues formally introduced the concept of apoptosis to describe a genetically regulated form of cell death essential for maintaining tissue homeostasis under physiological and pathological conditions (Kerr et al., 1972). As a canonical type I programmed cell death (PCD), apoptosis plays a critical role in development and disease, and its dysregulation is closely associated with cancer initiation, progression, and therapeutic resistance (Zhan et al., 2024; Galluzzi et al., 2018; Kashyap et al., 2021). Apoptosis is primarily executed through the intrinsic (mitochondrial and endoplasmic reticulum stress-related) and extrinsic (death receptor-mediated) pathways. Activation of these pathways leads to characteristic morphological changes, including cell shrinkage, membrane blebbing, and apoptotic body formation, which are subsequently cleared by phagocytes, thereby preventing inflammatory responses (Xu et al., 2019; Lossi, 2022).

The mitochondrial pathway represents a central apoptotic mechanism and is tightly regulated by Bcl-2 family proteins. Upon apoptotic stimulation, pro-apoptotic members such as Bax and Bak promote mitochondrial outer membrane permeabilization, resulting in the release of cytochrome c (Cyto-C) and subsequent formation of the apoptosome complex with Apaf-1. This complex activates caspase-9, which in turn triggers downstream effector caspases, including caspase-3, -6, and -7, ultimately leading to cellular dismantling (Shalini et al., 2015; Estaquier et al., 2012; Shakeri et al., 2017). In parallel, mitochondria also release caspase-independent apoptotic factors such as AIF and EndoG, as well as Smac/Diablo and HtrA2/Omi, which amplify apoptotic signaling by antagonizing inhibitor of apoptosis proteins (IAPs) (Joza et al., 2001; Tower, 2015).

Endoplasmic reticulum (ER) stress-induced apoptosis is initiated when prolonged disturbances in ER homeostasis activate the unfolded protein response (UPR). Under sustained stress conditions, UPR signaling shifts from adaptive responses toward apoptosis, primarily through PERK/eIF2α/ATF4/CHOP signaling and IRE1α-mediated activation of JNK, which cooperatively promote mitochondrial dysfunction and apoptotic commitment (Ishteyaque et al., 2023; Ibrahim et al., 2019; Guo et al., 2020; Kang et al., 2024).

The extrinsic apoptotic pathway is triggered by the engagement of death receptors, such as Fas, TNFR1/2, and DR4/5, with their corresponding ligands, including FasL, TNF-α, and TRAIL. Ligand-receptor binding induces the formation of the death-inducing signaling complex (DISC), leading to caspase-8 activation. Activated caspase-8 directly activates executioner caspases or indirectly amplifies apoptotic signaling through Bid cleavage and subsequent mitochondrial pathway activation (Thapa et al., 2020; Sessler et al., 2013; Waring and Müllbacher, 1999; Mahmood and Shukla, 2010).

**FIGURE 1:**
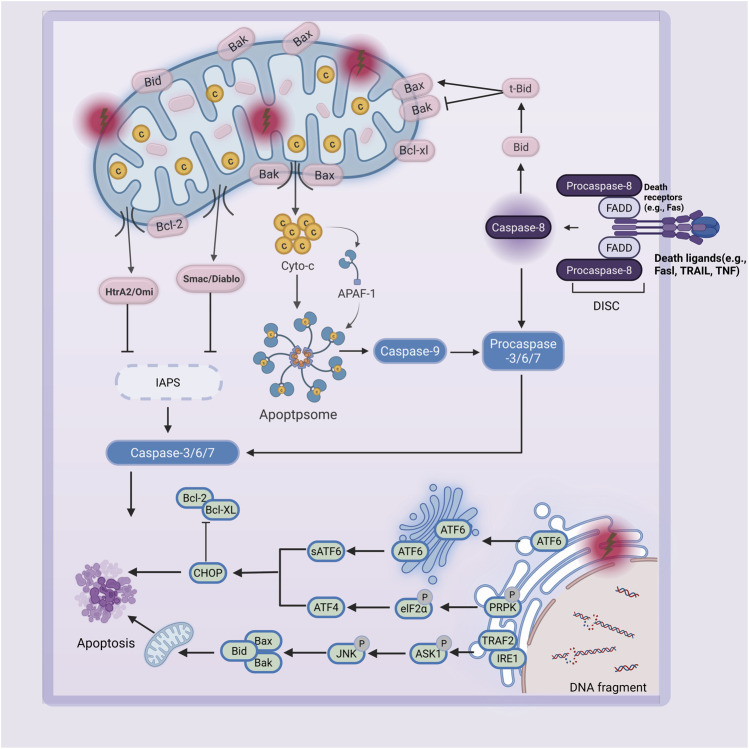
Three major regulatory pathways of apoptosis and their molecular mechanisms. Mitochondrial Pathway: Pro-apoptotic proteins such as Bax and Bak mediate the release of Cyto-C by forming transmembrane pores in the outer mitochondrial membrane. Cyto-C then binds to Apaf-1 to form the apoptosome, which activates caspase-9, further inducing apoptosis. Anti-apoptotic proteins such as Bcl-2 and Bcl-XL inhibit the activity of pro-apoptotic proteins, thereby preventing apoptosis. Death Receptor Pathway: Death ligands bind to their receptors, recruiting procaspase-8 through the FADD adaptor protein to form the DISC complex. This triggers the self-cleavage and activation of caspase-8, leading to apoptosis. Endoplasmic Reticulum Stress Pathway: Prolonged UPR activation triggers apoptosis via the IRE1α pathway, which activates the ASK1-JNK phosphorylation cascade. This process is further amplified through the PERK and ATF6 pathways, leading to the upregulation of CHOP and subsequent promotion of apoptosis. Apoptosis in osteosarcoma. Apoptosis in osteosarcoma is regulated by mitochondrial pathway, death receptor pathway, and ER stress pathway. These pathways collectively modulate osteosarcoma cell apoptosis.

Collectively, apoptosis is a highly coordinated PCD process orchestrated through mitochondrial, ER stress-related, and death receptor-mediated pathways (Figure 1). Aberrant regulation of these apoptotic signaling networks is a hallmark of osteosarcoma and contributes to tumor development, progression, and resistance to therapy, thereby providing a mechanistic foundation for therapeutic strategies aimed at restoring apoptotic sensitivity.

### 2.2 The role of apoptosis in osteosarcoma

In the mitochondria-mediated intrinsic apoptotic pathway, members of the Bcl-2 family, such as PUMA and Bim, interact with anti-apoptotic proteins Bcl-2 and Bcl-XL, thereby facilitating the activation of pro-apoptotic proteins Bax and Bak. This process results in a decrease in mitochondrial membrane potential (ΔΨm) and promotes the release of cytochrome c (Cyto-C) into the cytosol, which subsequently activates the caspase cascade and triggers apoptosis in osteosarcoma cells. The balance between pro- and anti-apoptotic Bcl-2 family members therefore plays a crucial role in regulating cell fate and represents a potential therapeutic target (Isoyama et al., 2023).

Several molecules have been reported to regulate osteosarcoma apoptosis through the mitochondrial pathway. For example, downregulation of ClC-5 promotes Bax translocation and mitochondrial apoptosis (Peng et al., 2020), while silencing cytochrome C1 (CYC1) enhances TRAIL-induced Cyto-C release and caspase-9 activation (Li et al., 2014). In addition, ectopic expression of FOXL1 and inhibition of the proto-oncogene c-Myc have been shown to promote mitochondrial apoptosis in osteosarcoma cells (Fan et al., 2020). The EZH2 inhibitor GSK343 can suppress both EZH2 and FBP1 expression, thereby indirectly affecting c-Myc signaling and promoting apoptosis (Rabenhorst et al., 2009; Koh et al., 2011). Activation of the c-Myc/caspase-2 axis further induces Bax activation, mitochondrial dysfunction, and Cyto-C release, leading to caspase-9/-3 activation (Cao et al., 2008).

The tumor suppressor p53 plays a central role in apoptosis regulation through both transcription-dependent and transcription-independent mechanisms (Han et al., 2001). Although PUMA and Bim are well-known p53 target genes, apoptosis in osteosarcoma can also occur through p53-independent mechanisms (Wang et al., 2022a). For instance, p14ARF activates caspase-9 to enhance cisplatin-induced apoptosis independently of p53 (Yuan et al., 2007), while miR-488 regulates apoptosis by directly targeting Bim under hypoxic conditions (Zhou et al., 2016). Other signaling pathways, including PI3K/AKT and Src-related signaling, also participate in regulating osteosarcoma apoptosis. Molecules such as RanBP9, TBRG4, FER1L4, and BUB1 have been reported to modulate apoptosis by influencing these pathways (Dai et al., 2016; Huang et al., 2020; Matsuura, 2019; Ma et al., 2019; Huang et al., 2021).

Endoplasmic reticulum (ER) stress also contributes to apoptosis regulation in osteosarcoma. The transcription factor ZBTB7A protects cells from ER stress-induced apoptosis by repressing lncRNA-GAS5 expression (Zhang et al., 2019), whereas LINC00629 inhibits ER stress-induced apoptosis through activation of the KLF4-LAMA4 pathway (Wang et al., 2022b).

In addition to the intrinsic pathway, the extrinsic death receptor pathway also plays an important role in osteosarcoma apoptosis. Activation of the Fas/FasL signaling pathway triggers caspase activation and apoptosis (Koshkina et al., 2020). Several regulators have been shown to modulate this pathway, including CLIC4, histone deacetylase (HDAC) inhibitors, and NF-κB-related signaling molecules such as TNFAIP1 and PKMYT1 (Suh et al., 2005; Watanabe et al., 2005; Li et al., 2019; Zhang C. et al., 2014; Lu et al., 2023).

Multiple additional proteins, including WWOX, HSP90, and Runx2, also influence apoptosis by regulating cytoskeletal organization and signaling pathways (Lo et al., 2015; Li J. et al., 2016; Shao et al., 2016; Liang et al., 2018; Eliseev et al., 2008). Furthermore, various molecules such as DHRS12, SOST, MAT2B, SOX4, SOX18, SATB1, ANXA3, PDCD10, CUL4A, TCTP, and CCT6A have been reported to participate in apoptosis regulation in osteosarcoma cells (Xu et al., 2020; Zou et al., 2017; Yuan et al., 2019a; Chen et al., 2018; Wu et al., 2016; Zhang H. et al., 2014; Zeng et al., 2019; Xu et al., 2023; Song et al., 2015; Shen et al., 2016; Zeng et al., 2022).

Non-coding RNAs (ncRNAs), including long non-coding RNAs (lncRNAs), microRNAs (miRNAs), and circular RNAs (circRNAs), are also important regulators of osteosarcoma apoptosis. By interacting with DNA, RNA, or proteins, these ncRNAs form complex regulatory networks that influence gene expression and signaling pathways involved in cell survival and death. Although numerous ncRNAs have been implicated in osteosarcoma apoptosis, they generally function through targeted regulatory mechanisms that ultimately determine cell fate (Figure 1).

Given the crucial role of apoptosis in restraining osteosarcoma progression and mediating therapeutic efficacy, strategies that restore or enhance apoptotic signaling may offer promising avenues for treatment. Therefore, natural compounds have attracted growing interest due to their ability to selectively induce apoptosis in tumor cells through diverse molecular mechanisms.

### 2.3 Natural products promote osteosarcoma cell apoptosis

Apoptosis, as a finely regulated form of PCD, has long been recognized as a fundamental mechanism for maintaining cellular homeostasis. However, when this process is disrupted, it often leads to the tumorigenesis. Resistance to apoptosis is a hallmark of cancer, enabling tumor cells to evade cell death and survive under adverse conditions, thereby driving cancer progression. As a strictly regulated form of PCD, apoptosis has become an important therapeutic target in cancer therapy. Tumor cells can be effectively removed by inducing apoptosis, thus inhibiting tumor growth and metastasis. In recent years, natural products have shown remarkable potential in the regulation of apoptosis-related molecules through a variety of pathways, triggering cell death and exerting powerful anti-tumor effects. In osteosarcoma, almost all studies on natural products are intricately linked to apoptosis. To provide a focused and high-impact perspective, this review will highlight several representative natural products derived from traditional medicinal plants, exploring their potential mechanisms and therapeutic value for osteosarcoma treatment through the regulation of apoptosis (Kazantseva et al., 2022). These include Oridonin from the traditional Chinese medicine *Rabdosia rubescens*, Wogonin from *Scutellaria baicalensis*, Triptolide from *Tripterygium wilfordii*, Evodiamine from *Evodia rutaecarpa*, Parthenolide from *Feverfew* (Tanacetum parthenium), Berberine from *Huanglian* (Coptis chinensis), Shikonin from *Lithospermum erythrorhizon* and Brusatol from Brucea javanica (Supplementary Table S1).

Together, these compounds exemplify the therapeutic promise of natural products in activating apoptotic pathways and offer valuable leads for the development of apoptosis-targeted therapies in osteosarcoma.

## 3 Natural products and autophagy in osteosarcoma

### 3.1 Overview of autophagy

Autophagy, derived from the Greek term meaning “self-eating,” is a conserved intracellular degradation process that maintains cellular homeostasis by recycling cytoplasmic components through the lysosomal pathway (Mizushima and Komatsu, 2011). In mammalian cells, autophagy is generally classified into macroautophagy, microautophagy, and chaperone-mediated autophagy, among which macroautophagy (hereafter referred to as autophagy) is the most extensively studied and biologically relevant in cancer (Yamamoto and Matsui, 2024).

Autophagy is dynamically regulated in response to diverse cellular stresses, including nutrient deprivation, oxidative stress, DNA damage, and protein aggregation (Nakatogawa, 2020). Mechanistically, the autophagic process proceeds through several coordinated stages: initiation, phagophore nucleation, autophagosome elongation, maturation, and lysosomal degradation (Galluzzi and Green, 2019). Under stress conditions, increased AMP/ATP ratios activate AMP-activated protein kinase (AMPK) and suppress mechanistic target of rapamycin complex 1 (mTORC1), thereby promoting activation of the ULK1 complex to initiate autophagy. Subsequent nucleation of the phagophore membrane is mediated by class III PI3K complexes containing Beclin-1, leading to phosphatidylinositol 3-phosphate (PI3P) production. During autophagosome elongation, microtubule-associated protein 1 light chain 3 (LC3) undergoes lipidation to form LC3-II, which facilitates membrane expansion and cargo sequestration. Mature autophagosomes then fuse with lysosomes to form autolysosomes, where the encapsulated contents are degraded and recycled.

**FIGURE 2:**
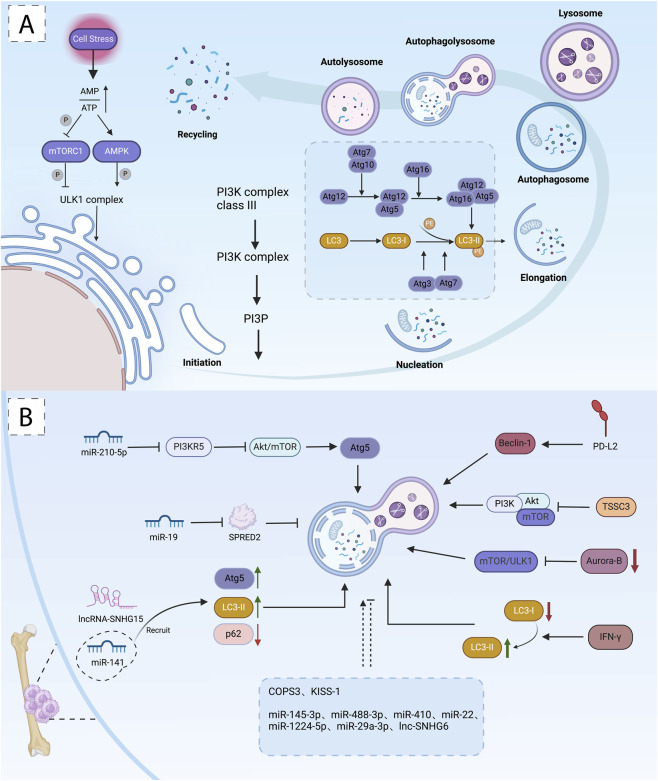
**(A)** Molecular mechanisms of autophagy. Autophagy is an evolutionarily conserved, dynamic process characterized by degradation. It involves several stages, including initiation, nucleation, elongation, maturation, and degradation. During this process, intracellular components and damaged organelles are progressively transported to lysosomes for breakdown and recycling. However, excessive activation of autophagy causes autophagic cell death. **(B)** Autophagy in osteosarcoma. Autophagy-related factors precisely regulate the autophagic process in osteosarcoma cells by modulating key signaling pathways (e.g., PI3K/Akt/mTOR and mTOR/ULK1), as well as by controlling the expression and activity of autophagy-related proteins (e.g., LC3, Beclin-1, p62, and Atg5).

Functionally, autophagy plays a context-dependent role in cellular fate determination. While basal autophagy supports cell survival by maintaining metabolic and organelle quality control, dysregulated or excessive autophagy can contribute to pathological conditions, including cancer. In osteosarcoma, accumulating evidence indicates that autophagy exerts both tumor-promoting and tumor-suppressive effects depending on disease stage, molecular context, and therapeutic pressure, highlighting its complex and dynamic role in tumor biology (Figure 2A).

### 3.2 The role of autophagy in osteosarcoma

Autophagy plays context-dependent roles in tumors and may exert pro-tumorigenic, anti-tumorigenic, or neutral effects (Almansa-Gómez et al., 2023). On the one hand, autophagy can support tumor progression by maintaining mitochondrial function and energy homeostasis, promoting cancer stem cell properties, and facilitating immune evasion (Miller and Thorburn, 2021; Ferro et al., 2020; Pan et al., 2013). On the other hand, autophagy may suppress tumorigenesis by removing damaged organelles and protein aggregates, thereby reducing oxidative stress and genomic instability (Gozuacik et al., 2017). As a form of programmed cell death, autophagic cell death (ACD) can also directly induce tumor cell death and inhibit tumor growth (Debnath et al., 2023).

Similar to other malignancies, autophagy plays a dual and dynamic role in osteosarcoma. During early tumor development, autophagy may help maintain cellular homeostasis while simultaneously providing metabolic support for rapid tumor growth. For example, programmed death ligand 2 (PD-L2) promotes autophagy through upregulation of Beclin-1, thereby enhancing invasion and metastasis of osteosarcoma cells (Ren et al., 2019). In addition, several microRNAs, including miR-210-5p and miR-19, activate autophagy through regulation of the AKT/mTOR pathway or its upstream regulators, thereby promoting osteosarcoma progression (Liu et al., 2020; Xie et al., 2020). The lncRNA SNHG15 can also stimulate autophagy by increasing Atg5 and LC3-II levels while decreasing p62 expression, ultimately accelerating tumor proliferation (Liu K. et al., 2017).

Under certain conditions, however, autophagy can exert tumor-suppressive effects. Interferon-γ (IFN-γ) has been shown to induce autophagy and apoptosis through LC3 conversion (Xu J. et al., 2021). In addition, several genes, including COPS3 and KISS-1, can activate autophagic pathways and suppress the malignant phenotype of osteosarcoma cells (Zhang et al., 2018; Yin et al., 2017). Various non-coding RNAs, such as miR-145-3p, miR-488-3p, and miR-410, have also been reported to regulate autophagy-related signaling pathways and inhibit tumor progression (Wu et al., 2018; Yun et al., 2024a; Chen et al., 2017; Guo et al., 2014; Jin et al., 2020; Qi et al., 2022; Zhu X. et al., 2019).

Autophagy also plays complex roles in osteosarcoma metastasis. In some contexts, activation of autophagy suppresses metastasis. For instance, knockdown of SAR1A enhances autophagic activity and significantly inhibits pulmonary metastasis (Zhan et al., 2022), while inhibition of Aurora-B induces autophagy through the mTOR/ULK1 pathway and suppresses metastatic potential (Wu X. et al., 2020). The tumor suppressor gene TSSC3 similarly inhibits metastasis by promoting autophagy (Zhao et al., 2018). However, autophagy can also promote metastasis under certain molecular conditions. Overexpression of miR-210-5p or activation of the VCP-mediated ERK/NF-κB/Beclin-1 signaling pathway enhances autophagy and apoptosis resistance, thereby facilitating metastatic progression (Liu et al., 2020; Long et al., 2019).

Chemoresistance remains a major challenge in osteosarcoma treatment (Rosen et al., 1976), and accumulating evidence indicates that autophagy plays an important role in this process. Autophagy often functions as a survival mechanism that enables tumor cells to withstand chemotherapeutic stress. For example, the HSP90 isoform HSP90AA1 promotes autophagy through the PI3K/Akt/mTOR pathway and contributes to chemoresistance (Xiao et al., 2018). Similarly, GFRA1 enhances cisplatin resistance by activating autophagy (Kim et al., 2017), while the transcription factor FOXM1 drives methotrexate resistance via an HMMR/ATG7-dependent autophagic pathway (Wang L. et al., 2024). In addition, COPS3 promotes chemoresistance through FOXO3-mediated autophagy activation (Niu et al., 2023). Conversely, suppression of autophagy can also contribute to drug resistance; for instance, ANXA2 and Rac1 inhibit autophagy and increase cisplatin resistance (Pan et al., 2023).

Non-coding RNAs are important regulators of autophagy-mediated chemoresistance in osteosarcoma. Several miRNAs, including miR-22, miR-30a, miR-101, and miR-143, inhibit autophagy and thereby reduce chemoresistance (Meng et al., 2020; Xu et al., 2016; Chang Z. et al., 2014; Zhou et al., 2015a; Li Y. et al., 2016). In contrast, miR-193b, miR-140-5p, and miR-155 promote autophagy and enhance drug resistance (Dong et al., 2019; Meng et al., 2017; Chen L. et al., 2014). According to the competing endogenous RNA (ceRNA) hypothesis, certain lncRNAs function as molecular sponges for these miRNAs to regulate autophagy-related genes. For example, Sox2OT-V7 and SNHG16 promote doxorubicin resistance by activating autophagy through miRNA-mediated regulatory networks (Zhu et al., 2020; Liu et al., 2019) (Figure 2B).

Overall, the dual role of autophagy suggests that precise regulation of autophagic flux may represent a promising therapeutic strategy. Maintaining autophagy at an optimal level at the “crossroads of life and death” may determine the fate of osteosarcoma cells. Accordingly, natural products capable of modulating autophagy have attracted increasing attention as potential therapeutic agents for osteosarcoma.

### 3.3 Natural products bidirectionally regulate autophagy and ultimately promote osteosarcoma cell death

Autophagy, a critical cellular process for maintaining homeostasis, is tightly regulated by multiple signaling pathways, with mTOR signaling functioning as a central regulatory node. mTOR serves both as a “nutrient state sensor” and a regulatory switch in autophagy. Recent studies have highlighted the potential of natural products to modulate these pathways and thereby induce autophagy, representing promising therapeutic strategies for osteosarcoma.

Soy isoflavones (SI), an active ingredient derived from *soybeans*, inhibit osteosarcoma growth by suppressing autophagy via the AKT/mTOR pathway, thereby inhibiting tumor proliferation, migration, and invasion (Zheng et al., 2024). Similarly, Betulin, a compound extracted from the outer bark of white and yellow birch trees, promotes autophagy by inhibiting mTOR and upregulating LC3-II expression (Lin et al., 2026). Butein, a *chalcone polyphenol*, also induces autophagy through AKT/mTOR inhibition and upregulation of LC3-II (Zhang et al., 2026). Andrographolide (AG), a lactone diterpenoid from *Andrographis paniculata*, regulates autophagy through multiple mechanisms to influence osteosarcoma growth and metastasis. Mechanistic studies indicate that AG induces autophagy by inhibiting the PI3K/Akt/mTOR pathway while activating the JNK pathway, thereby promoting autophagic cell death and reducing osteosarcoma malignancy (Liu Y. et al., 2017). Imperatorin, a bioactive compound extracted from the traditional Chinese medicine *Angelica sinensis*, inhibits the PTEN-PI3K-AKT-mTOR/p21 signaling pathway and upregulates ULK1, Atg5, and LC3-II/LC3-I, thereby promoting autophagic cell death in osteosarcoma cells (Lv et al., 2021). Cardamom also promotes autophagy by inhibiting the mTOR pathway (Li et al., 2025). Additionally, Baicalin regulates the autophagy via key mediators of increased Ca^2+^ and ROS levels. This effect can be reversed by chelating Ca^2+^ with BAPTA-AM or scavenging ROS with NAC (Pang et al., 2022).

The JNK pathway, a key regulator of stress-induced autophagy, is activated by various internal and external stimuli, including oxidative stress, cell damage, and inflammation (Zhou et al., 2015b). Many natural products modulate autophagy by targeting the JNK-related signaling pathway. For instance, Curcin C, a natural product from the cotyledons of post-germinated *Jatropha curcas seeds*, promotes autophagy by inducing ROS production, leading to activation of the JNK signaling pathway (Wang F. et al., 2026). Similarly, the natural bioactive compounds Peiminine from Fritillaria thunbergii (Yu et al., 2021), Polyphyllin VI from *Paris polyphylla* (Yuan et al., 2019b), Celastrol from the traditional medicine “Thunder of God Vine” (Li et al., 2015), and Erianin from *Dendrobium chrysotoxum* (Wang et al., 2016) exert similar effects. They promote autophagic cell death in osteosarcoma via the ROS/JNK signaling pathway, thereby suppressing tumor progression.

FOXO transcription factors are key reg ulators of autophagy through transcriptional control of genes involved in diverse cellular processes. Cirsiliol, a plant-derived flavonoid with antitumor properties, modulates osteosarcoma cell behavior by inhibiting AKT phosphorylation, thereby activating FOXO1-mediated transcription (Luo et al., 2023). Extracellular signal-regulated kinase 1/2 (ERK1/2), a key MAPK family member, is another critical regulator of autophagy. Dendropanoxide (DP), a natural product from the leaves and stems of *Dendropanax morbifera*, effectively modulates ERK1/2 signaling, exhibiting high efficacy and low toxicity, and induces autophagy by activating the ERK1/2 pathway (Lee et al., 2013). Honokiol (HNK), a biphenolic compound extracted from the *Magnolia* tree, also activates the ROS/ERK1/2 signaling pathway to promote autophagy (Huang et al., 2018).

Nuclear protein 1 (NUPR1), a recently characterized nuclear protein involved in diverse biological processes, promotes tumor progression and metastasis in multiple malignancies (Emma et al., 2016; Santofimia-Castaño et al., 2019). Recent studies reveal that NUPR1 modulates cell proliferation and survival by regulating autophagic flux. Quercetin induces autophagic cell death in osteosarcoma cells by activating the NUPR1 pathway (Wu B. et al., 2020).

Triptolide, a bioactive compound from Tripterygium wilfordii, modulates autophagic flux and induces autophagy by inhibiting the Wnt/β-catenin signaling pathway (Li et al., 2018). Beyond signaling pathway modulation, direct interaction with autophagy-related proteins is another key mechanism through which natural products regulate autophagy. Numerous studies demonstrate that various natural products directly regulate autophagy-related proteins, including Escin (Zhu et al., 2017), Fraxinellone (He et al., 2021), Norcantharidin (Mei et al., 2019), Polydatin (Jiang C. et al, 2020), Protodioscin (Huang et al., 2023), Naringenin (Lee et al., 2022), Licochalcone A (Shen et al., 2019), Licochalcone B (Huang and Jin, 2022), Anthocyanins (Choe et al., 2012), Polyphyllin VII (Li et al., 2021), Cinobufagin (Ma et al., 2016), Carnosol (Lo et al., 2026), Tanshinone IIA (Ma et al., 2026), 11-O-galloyl bergenin (Park, 2026), Neohesperidin (Wang, 2026), Ginseng polysaccharide (Zhang, 2026), and Bufalin (Zheng et al., 2026). By directly interacting with these proteins or regulating their expression levels, these natural products affect the formation, maturation and degradation of autophagosomes. This ultimately promotes the autophagy to inhibit tumor cell growth, migration and metastasis, thereby demonstrating great anti-tumor potential.

The regulatory effects of natural products on the autophagy process are bidirectional. In most cases, natural products promote autophagy, influencing osteosarcoma cell growth and survival, while also attenuating their malignancy through autophagic cell death. However, some natural products exhibit the opposite regulatory effect. For example, Panax notoginseng saponins inhibit autophagy. Notably, this effect does not impair their antitumor efficacy (Han et al., 2021). Instead, they effectively induce apoptosis in osteosarcoma cells, compensating for any potential reduction in antitumor activity due to autophagy inhibition (Supplementary Table S2).

As previously noted, natural products can modulate critical molecular regulators to shift autophagy from a “pro-survival” to a “pro-death” mode. For instance, they may inhibit PI3K/Akt signaling or directly suppress mTOR activity, thereby relieving ULK1 and Beclin-1 from inhibitory regulation. This initiates autophagosome formation and drives cells toward autophagic cell death. Some natural products also directly upregulate ULK1, enhancing autophagy nucleation and promoting autophagic cell death. Additionally, natural products can modulate other molecular regulators, including Beclin-1, JNK, ERK1/2, and FOXO, to facilitate osteosarcoma cell death.

Importantly, the regulation of autophagy by natural products exhibits dose dependency and cell type specificity. For example, under the same 48-h treatment conditions, U-2 OS cells were the most sensitive to Curcin C in activating JNK and triggering autophagic cell death, with an IC50 of 0.041 μM, which is significantly lower than that of MG-63 (0.2484 μM) and HOS (0.2698 μM) cells (Wang F. et al., 2026). This suggests that although Curcin C can induce autophagy via the ROS/JNK axis and downstream switches such as Beclin-1 in all 3 cell lines, differences in genetic background and molecular expression patterns result in varying levels of responsiveness, thereby leading to distinct growth-inhibitory effects. Furthermore, U-2 OS cells have shown greater sensitivity to soy isoflavones, displaying most significantly inhibited cell viability compared to Saos-2 and MG63 cells (Zheng et al., 2024). Thus, different osteosarcoma cell lines have significant differences in their responses to the same natural product.

In summary, natural products regulate autophagy in osteosarcoma through diverse mechanisms and molecular switches, exhibiting context-dependent and cell type-specific effects. Their ability to either promote or inhibit autophagy highlights the therapeutic complexity and precision required for targeting this pathway. These outcomes underscore the importance of further mechanistic studies in multiple osteosarcoma models to optimize the clinical potential of autophagy-targeting natural compounds.

## 4 Natural products and ferroptosis in osteosarcoma

### 4.1 Overview of ferroptosis

Ferroptosis, first defined by Dixon et al., in 2012, is a distinct form of regulated cell death characterized by iron-dependent lipid peroxidation and excessive accumulation of reactive oxygen species (ROS), and is mechanistically and morphologically different from apoptosis and autophagy (Dixon et al., 2012). Increasing evidence indicates that ferroptosis is a tightly regulated process governed by iron metabolism, lipid peroxidation, and redox homeostasis.

Dysregulation of intracellular iron metabolism is a key driver of ferroptosis. Circulating Fe^3+^ is transported into cells via transferrin receptor 1 (TfR1) and subsequently reduced to Fe^2+^, which enters the labile iron pool (LIP). Excessive accumulation of Fe^2+^ promotes ROS generation through the Fenton reaction, thereby amplifying oxidative stress and sensitizing cells to ferroptotic death (Liu et al., 2022; Tang D. et al., 2021).

Lipid peroxidation of polyunsaturated fatty acid (PUFA)-containing phospholipids represents another hallmark of ferroptosis. Enzymes such as acyl-CoA synthetase long-chain family member 4 (ACSL4) and lysophosphatidylcholine acyltransferase 3 (LPCAT3) facilitate the incorporation of PUFAs into membrane phospholipids, which are highly susceptible to lipoxygenase-mediated oxidation, leading to the accumulation of lethal lipid peroxides (Ma X. et al., 2024). Multiple signaling pathways, including p53-related axes (p53/SAT1/ALOX15 and p53/SLC7A11), glutamate metabolism, and the p62/Keap1/Nrf2 pathway, further modulate ferroptotic sensitivity by regulating lipid metabolism and antioxidant defenses (Li et al., 2022; Ma X. et al., 2024; Yang et al., 2022).

**FIGURE 3:**
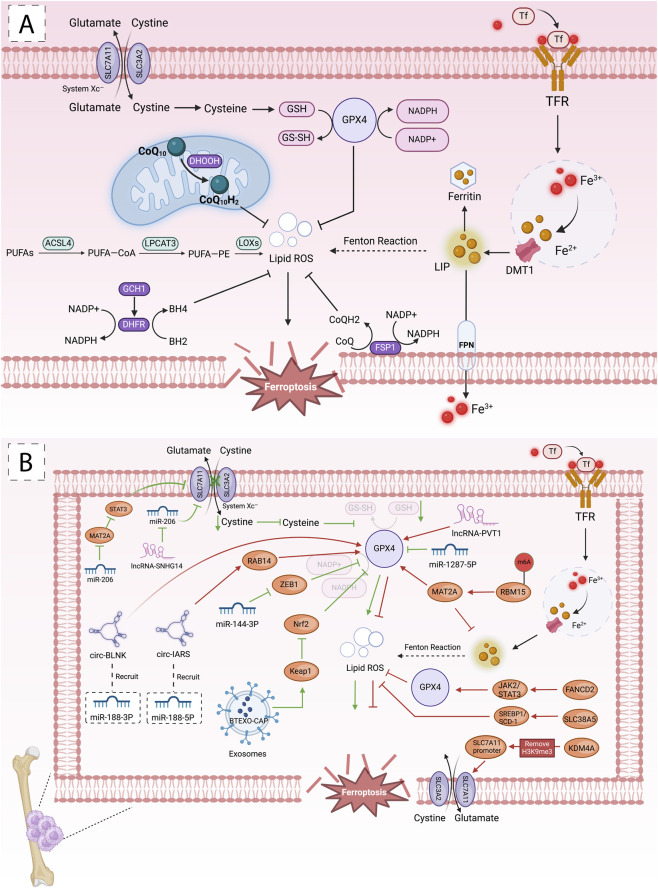
**(A)** Molecular mechanisms of ferroptosis. Ferroptosis arises from the interplay of toxic effects caused by intracellular free iron accumulation, decreased glutathione levels, and lipid peroxidation damage to bio membranes. Cells can resist this process through multiple defense systems, including the SystemXc^−^-dependent glutathione synthesis and GPX4 antioxidant pathway, the FSP1-mediated NADPH-CoQ10 reduction system axis, the GCH1/DHFR/BH4 axis, and the DHODH/CoQ10 axis. These protective mechanisms collaboratively regulate cellular redox balance, effectively preventing iron-dependent cell death. **(B)** Ferroptosis in osteosarcoma. Ferroptosis-related factors control lipid peroxidation levels in osteosarcoma cells by regulating iron metabolism, ferroptosis-related axes, and key ferroptosis protein expression, thereby modulating ferroptosis.

Cells possess several protective systems to counteract ferroptosis. The cystine/glutamate antiporter system Xc^−^ (SLC7A11/SLC3A2) supports glutathione (GSH) synthesis, which serves as a critical cofactor for glutathione peroxidase 4 (GPX4) to detoxify lipid peroxides. In addition, emerging ferroptosis-suppressive pathways, including the ferroptosis suppressor protein 1 (FSP1)/NADPH/coenzyme Q10 (CoQ10) axis, the GCH1/DHFR/tetrahydrobiopterin (BH4) axis, and the dihydroorotate dehydrogenase (DHODH)/CoQ10 axis, provide alternative antioxidant defense mechanisms independent of GPX4 (Soula et al., 2020; Guo et al., 2024) (Figure 3A).

### 4.2 The role of ferroptosis in osteosarcoma

Iron overload is a prerequisite for ferroptosis. RNA Binding Motif Protein 15 (RBM15), a gene implicated in multiple diseases including osteosarcoma, is upregulated in osteosarcoma tissues and cells. RBM15 promotes methionine adenosyltransferase 2 alpha (MAT2A) expression via N6-methyladenosine (m6A) modification. It also reduces intracellular Fe^2+^ levels and upregulates GPX4, thereby inhibiting ferroptosis (Huang et al., 2025).

GPX4 is a central regulator of ferroptosis, and its activity critically determines ferroptotic responses in osteosarcoma cells. Increasing evidence indicates that non-coding RNAs play an important role in regulating GPX4 expression. For instance, miR-1287-5p promotes ferroptosis by directly targeting GPX4 (Xu Z. et al., 2021). Conversely, certain circular RNAs, such as circ-BLNK and circ-IARS, function as molecular sponges for specific miRNAs, thereby upregulating GPX4 expression and suppressing ferroptosis in osteosarcoma cells (Li et al., 2023b; Li Y. et al., 2024). Similarly, lncRNA-PVT1 inhibits ferroptosis through activation of the STAT3/GPX4 signaling pathway (Li G. et al., 2024). In contrast, miR-144-3p promotes ferroptosis by suppressing ZEB1 and regulating ferroptosis-related proteins including GPX4, ACSL4, and SLC7A11 (Jiang et al., 2023). BT-EXO-CAP also inhibits the nuclear translocation of Nrf2, contributing to ferroptotic cell death. Suppression of the Nrf2/GPX4 signaling pathway via Keap1 activation is critical for BT-EXO-CAP-induced ferroptosis in osteosarcoma cells. Moreover, Nrf2 overexpression effectively reverses BT-EXO-CAP-induced Fe^2+^ accumulation, lipid peroxidation, and GSH depletion (Chen et al., 2023).

SLC7A11 is another key protein that protects cells from ferroptosis. Suppression of the Nrf2/SLC7A11/GPX4 axis has been shown to trigger ferroptosis in osteosarcoma cells (Qin et al., 2025). lnc-SNHG14 inhibits ferroptosis in osteosarcoma cells through the miR-206/SLC7A11 axis (Li et al., 2023c). Moreover, miR-26b-5p inhibits the MAT2A expression and triggers ferroptosis in osteosarcoma cells by increasing intracellular ferrous iron levels and suppressing the STAT3/SLC7A11 axis (Xia et al., 2023). Lysine-specific demethylase 4A (KDM4A), an important epigenetic regulator, inhibits ferroptosis by removing the H3K9me3 mark from the promoter region of SLC7A11, thereby activating its transcription (Chen et al., 2021). Moreover, activation of the system Xc^−^/GPX4 axis has been linked to ferroptosis resistance in osteosarcoma. Phosphoserine aminotransferase 1 (PSAT1) enhances SLC7A11/GPX4 signaling, thereby suppressing lipid peroxidation and ferroptotic cell death in osteosarcoma cells (Wang P. et al., 2025).

In addition to these core pathways, several signaling networks have been reported to regulate ferroptosis in osteosarcoma. For example, FANCD2 suppresses ferroptosis by modulating the JAK2/STAT3 pathway (Li and Liu, 2023), while SLC38A5 inhibits ferroptosis through glutamine-mediated activation of the PI3K/AKT/mTOR pathway (Huang X. et al., 2024). Moreover, the METTL1/pri-miR-26a/FTH1 axis highlights the role of m7G RNA methylation in regulating ferroptosis and chemoresistance in osteosarcoma, suggesting that RNA methylation reprogramming may represent a potential therapeutic strategy (He et al., 2024) (Figure 3B).

Overall, these findings reveal that ferroptosis is tightly regulated by iron metabolism, antioxidant systems such as GPX4, and multiple signaling pathways in osteosarcoma. Based on these mechanistic insights, natural products capable of modulating ferroptosis have attracted increasing attention as potential therapeutic agents for osteosarcoma.

### 4.3 Natural products promote osteosarcoma cell ferroptosis

As noted above, ferroptosis acts as a pivotal cell death mechanism in osteosarcoma, particularly in the context of photodynamic therapy (PDT). PDT induces ROS to trigger cancer cell apoptosis, but its efficacy is often compromised by the hypoxic tumor microenvironment (TME). HIF-1α, a major mediator of hypoxic TME, is upregulated under low-oxygen conditions and promotes tumor progression. To overcome this limitation, researchers have developed a nanoplatform (CI@HSA NPs) for synergistic treatment under near-infrared (NIR) irradiation, offering a novel strategy to enhance the cytotoxicity of PDT. Their studies have identified that CI@HSA NPs, incorporating the natural compound Capsaicin, activate the transient receptor potential vanilloid 1 (TRPV1) channel, leading to an increase in intracellular Ca^2+^ concentration. This activation inhibits the Nrf2/GPX4 pathway, promoting ferroptosis while simultaneously suppressing HIF-1α expression to reduce oxygen consumption and alleviate hypoxia in the TME. These effects significantly enhance the therapeutic efficacy of PDT in osteosarcoma. Mechanistic studies have further demonstrated that these outcomes are mediated through activation of MAPK (ERK, JNK, and P38) and PI3K/AKT signaling pathways (Wang Y. et al., 2024).

Casticin, another natural compound, upregulates HMOX1, LC3, and NCOA4, resulting in Fe^2+^ accumulation, elevated ROS levels, and ferroptosis induction. This consequently suppresses osteosarcoma cell growth and metastasis. Notably, Casticin also activates the MAPK signaling pathway, potentially contributing to ferroptosis induction (Jiwa et al., 2024). Similarly, Theaflavin-3,3′-digallate (TF3), a natural tea polyphenol, induces ferroptosis in osteosarcoma cells via the MAPK pathway (He et al., 2022). Nevertheless, the precise role of MAPK in ferroptosis remains unclear and warrants further investigation.

Gambogenic acid (GNA), a bioactive compound isolated from *Garcinia*, has recently attracted attention for its anti-cancer properties (Wang et al., 2021). In osteosarcoma, GNA induces oxidative stress through multiple mechanisms, including marked increases in ROS production and lipid peroxidation, accompanied by depletion of intracellular GSH, thereby weakening cellular defense against ferroptosis. GNA also downregulates SLC7A11 and GPX4 expression, further sensitizing osteosarcoma cells to ferroptosis. Furthermore, GNA upregulates p53, activating the p53/SLC7A11/GPX4 axis, a critical regulatory pathway of ferroptosis. This signaling cascade disrupts lipid metabolism and triggers ferroptosis, exerting potent cytotoxic effects on osteosarcoma cells (Liu et al., 2023).

Shikonin, an active compound extracted from the root of ZiCao, induces ferroptosis in osteosarcoma cells by increasing Fe^2+^, ROS, and lipid peroxidation. Mechanistically, Shikonin promotes ubiquitin-mediated degradation of Nrf2, suppressing xCT and GPX4 expression (Huang R. et al., 2024), and also triggers ferroptosis via the HIF-1α/HO-1 axis, where mitochondrial ROS indirectly regulate HIF-1α (Lu et al., 2024). Baicalin, a flavonoid from the dried roots of Scutellaria baicalensis, induces ferroptosis primarily via the Nrf2/xCT/GPX4 axis (Wen et al., 2023). By activating Nrf2, Baicalin upregulates xCT, leading to Fe^2+^ accumulation, ROS production, and malondialdehyde (MDA) generation, while further enhancing oxidative stress by reducing the GSH/GSSG ratio. While both Baicalin and Shikonin promote ferroptosis through Fe^2+^ and ROS accumulation, Shikonin additionally activates Nrf2 ubiquitin degradation and the HIF-1α/HO-1 axis, highlighting distinct mechanistic pathways and potential for combinatorial strategies. Curcumin, a bioactive compound from Curcuma longa L., induces ferroptosis via the Nrf2/GPX4 axis, enhancing GPX4 expression and oxidative stress to drive lipid peroxidation and iron overload (Yuan et al., 2024). Similarly, Curculigoside promotes ferroptosis by increasing Fe^2+^ accumulation and ROS generation while downregulating GPX4 (Ma et al., 2025).

Bavachin, a natural flavonoid, shows an important role in inhibiting the malignant proliferation of osteosarcoma. Recent studies have shown that Bavachin induces ferroptosis in osteosarcoma cells by inhibiting GPX4, SLC7A11 and p-STAT3 expression and increasing P53 expression (Luo et al., 2021). Oridonin, a biologically active compound isolated from *Rabdosia rubescens*, also has anti-tumor activity (Liu et al., 2021). In osteosarcoma studies, Oridonin has been confirmed to promote cellular ferroptosis by downregulating SLC7A11, GPX4 and FTH1 expression, while upregulating ACSL4 expression (Zhang et al., 2024). In addition, Sulforaphane can promote the degradation of SLC7A11 in lysosomes, triggering ferroptosis (Zou et al., 2025). (Supplementary Table S3).

Various natural products selectively regulate ferroptosis-related pathways, providing novel strategies to inhibit osteosarcoma progression. These findings expand our understanding of ferroptosis regulation and highlight potential avenues for combination therapy in osteosarcoma.

## 5 Natural products and pyroptosis in osteosarcoma

### 5.1 Overview of pyroptosis

Pyroptosis is a distinct form of programmed cell death (PCD) characterized by membrane pore formation, cell swelling, membrane rupture, and the release of pro-inflammatory intracellular contents. It was first described in 2001 in Salmonella-infected macrophages as a cell death phenotype morphologically and mechanistically distinct from classical apoptosis (Ketelut-Carneiro et al., 2022; Cookson and Brennan, 2001).

Although initially defined based on morphological features, the molecular basis of pyroptosis was not fully elucidated until the identification of gasdermin D (GSDMD) as the key pore-forming effector protein in 2015 (Shi et al., 2015). Subsequently, the Cell Death Nomenclature Committee formally redefined pyroptosis in 2018 as a gasdermin-mediated form of regulated cell death (Galluzzi et al., 2018). To date, members of the gasdermin family (GSDMA–GSDME) have been recognized as the ultimate executors of pyroptosis, with their N-terminal fragments forming membrane pores upon proteolytic cleavage.

Pyroptosis can be initiated through multiple upstream signaling pathways, including classical and non-classical inflammasome pathways, apoptosis-related caspase-mediated pathways, and granzyme-mediated pathways. In the classical inflammasome pathway, pattern recognition receptors such as NLRP family members, AIM2, and pyrin assemble inflammasomes that activate caspase-1, leading to the cleavage of GSDMD and the maturation of interleukin-1β (IL-1β) and interleukin-18 (IL-18) (Kovacs and Miao, 2017; You et al., 2022; Dubyak et al., 2023; He et al., 2016). In the non-classical pathway, caspase-4/5/11 directly sense intracellular lipopolysaccharide (LPS) and cleave GSDMD, triggering pyroptosis and secondarily activating inflammasomes through ion fluxes (Wang et al., 2017; Wang et al., 2020; Wei et al., 2022).

**FIGURE 4:**
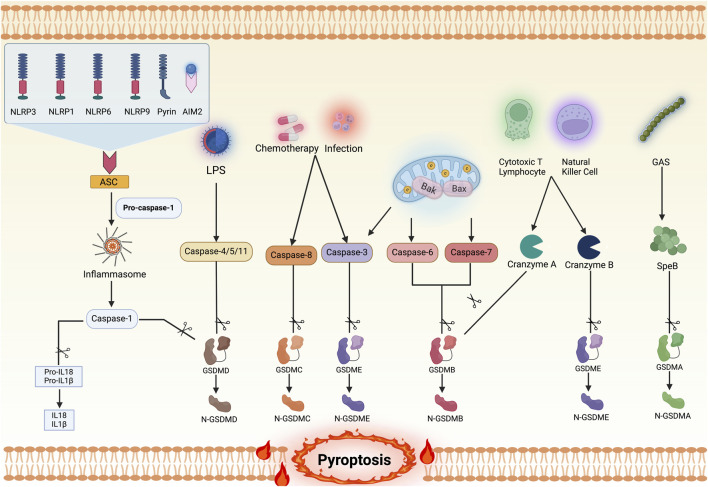
Molecular mechanisms of pyroptosis. Pyroptosis primarily occurs through the classical and non-classical pathways. In the classical (classical inflammasome) pathway, inflammasome sensors (e.g., NLRP3) are activated by danger signals (e.g., PAMPs/DAMPs), recruiting procaspase-1 via the adaptor protein ASC to form the inflammasome complex. Activated caspase-1 cleaves GSDMD to generate pore-forming N-GSDMD, which disrupts the cell membrane, while simultaneously processing pro-IL-1β and pro-IL-18 into mature cytokines (IL-1β and IL-18) to amplify inflammation and drive pyroptosis. The non-classical pathways include (a) the non-classical inflammasome pathway, where LPS directly activates caspase-4/5/11 (human: caspase-4/5; murine: caspase-11) to cleave GSDMD; (b) caspase-mediated pathways, where caspase-3 cleaves GSDME and caspase-8 cleaves GSDMC; (c) granzyme-mediated pathway, where granzyme A and caspase-6/7 target GSDMB, granzyme B cleaves GSDME, and *streptococcal protease SpeB* directly cleaves GSDMA-all culminating in pyroptosis.

In addition to inflammasome-dependent mechanisms, apoptosis-associated caspases and immune cell-derived granzymes can also induce pyroptosis by cleaving different gasdermin family members. Caspase-3 and caspase-8 can convert apoptosis into pyroptosis by cleaving GSDME or GSDMC under specific stress conditions, including chemotherapy (Li et al., 2020; Jiang M. et al., 2020; Hou et al., 2020). Similarly, granzyme A and granzyme B released from cytotoxic lymphocytes directly cleave GSDMB and GSDME, respectively, providing an immune-mediated route to pyroptotic cell death (Zhang et al., 2020; Zhou Z. et al., 2020; Chowdhury and Lieberman, 2008). More recently, pathogen-derived proteases have also been shown to activate gasdermins, as exemplified by Streptococcal pyrogenic exotoxin B-mediated cleavage of GSDMA, further expanding the mechanistic landscape of pyroptosis (Wang M. et al., 2026) (Figure 4).

Given its unique inflammatory characteristics, pyroptosis has attracted growing interest for its role in tumorigenesis. Although extensively studied in other malignancies, its functional implications in osteosarcoma remain poorly understood. This section highlights the emerging roles of pyroptosis in osteosarcoma progression and therapeutic strategies.

### 5.2 The role of pyroptosis in osteosarcoma

Pyroptosis is a pro-inflammatory form of programmed cell death that can stimulate anti-tumor immune responses and therefore represents a potential therapeutic strategy for cancer. Although studies investigating pyroptosis in osteosarcoma remain relatively limited, increasing attention has been paid to its potential role in prognosis and tumor progression.

Several studies have constructed prognostic models based on pyroptosis-related genes or non-coding RNAs in osteosarcoma. For example, one study identified six pyroptosis-associated genes (BAK1, CASP5, CASP6, GPX4, GZMA, and CHMP4C) and established a prognostic scoring system termed the PRS score (Zhang et al., 2021). Other analyses have similarly reported that pyroptosis-related lncRNA signatures are closely associated with the tumor microenvironment and clinical outcomes in osteosarcoma patients (Bu et al., 2021; Yang et al., 2023). In addition, hypoxia within the tumor microenvironment has been suggested to influence pyroptosis and immune responses, which may affect chemoresistance and disease prognosis (Hu et al., 2022).

Mechanistically, several molecules have been reported to regulate pyroptosis in osteosarcoma cells. For instance, CHMP4C has been identified as a potential biomarker highly expressed in osteosarcoma and associated with tumor progression (Zhang et al., 2022). In contrast, DOT1L has been shown to induce pyroptosis in Saos-2 osteosarcoma cells through activation of the STING–NLRP3 signaling pathway, leading to caspase-1 activation and gasdermin D cleavage. *In vivo* studies further demonstrated that modulation of this pathway suppresses tumor growth in xenograft models (Hu, 2025).

Recent advances in nanotechnology and biomaterials have also enabled the development of novel therapeutic strategies that induce pyroptosis in osteosarcoma. For example, biomimetic nanoplatforms integrating macrophage membranes, photosensitizers, and ROS-generating polymers can trigger pyroptosis through photothermal effects and oxidative stress while simultaneously promoting bone regeneration (Ma Q. et al., 2024). Similarly, dual-metal nanoplatforms and near-infrared photothermal agents have been reported to induce pyroptosis while enhancing anti-tumor immune responses and facilitating bone repair (Shi et al., 2023; Liu K. et al., 2024).

Overall, these findings highlight the therapeutic potential of targeting pyroptosis in osteosarcoma. However, the clinical translation of many emerging strategies remains limited by issues such as delivery efficiency, safety, and production cost. In this context, natural products have attracted increasing attention as potential modulators of pyroptosis, and their roles in osteosarcoma will be discussed in the following section.

### 5.3 Natural products promote osteosarcoma cell pyroptosis

As a pro-inflammatory form of PCD, pyroptosis is emerging as a promising strategy to trigger non-apoptotic cell death in cancer therapy. Moreover, pyroptosis has shown considerable potential as a prognostic indicator in osteosarcoma.

Recent studies have discovered that Dioscin, a steroid saponin extracted from medicinal plants, significantly inhibits human osteosarcoma growth (Ding et al., 2020). This natural product not only exhibits inhibitory effects on osteosarcoma cell proliferation but also induces G2/M phase cell cycle arrest and apoptosis *in vitro*. Morphologically, Dioscin triggers characteristic apoptotic changes in osteosarcoma cells, including cell shrinkage, chromatin condensation, and nuclear fragmentation. Mechanistic analysis revealed that the caspase-3-GSDME-N axis plays a critical role in Dioscin-induced apoptosis.

The activation of caspase-3 leads to the cleavage of GSDME, generating the GSDME-N fragment. This fragment penetrates the cell membrane, forming pores that ultimately induce pyroptosis. The formation of pores in the cancer cell membrane by GSDME is a critical mechanism of pyroptosis. Therefore, the anticancer potential of Dioscin lies not only in its ability to inhibit osteosarcoma cell proliferation and induce apoptosis but also in its capacity to promote pyroptosis through the GSDME pathway. Although current studies on natural products inducing pyroptosis in osteosarcoma cells mainly focus on Dioscin, which plays a critical role in triggering pyroptosis, highlighting its great potential as a therapeutic agent. Nevertheless, existing evidence offers encouraging perspectives for applying natural product-mediated pyroptosis pathways in osteosarcoma therapy (Supplementary Table S4).

Despite the growing interest in pyroptosis as an inflammatory form of programmed cell death, its mechanistic roles in osteosarcoma remain incompletely understood. Moreover, experimental evidence supporting natural product–induced pyroptosis in osteosarcoma is still limited. Future studies employing more rigorous mechanistic investigations, particularly in immune-competent models, will be essential to determine whether pyroptosis predominantly exerts tumor-suppressive or pro-tumorigenic effects in osteosarcoma.

## 6 Natural products and necroptosis in osteosarcoma

### 6.1 Overview of necroptosis

Necroptosis is a regulated form of non-apoptotic cell death that was first described in 2005 by Degterev et al., who identified a specific signaling cascade inhibited by the small molecule necrostatin-1 (Degterev et al., 2005). This discovery challenged the traditional view of necrosis as a passive and unregulated process and established necroptosis as an active, genetically controlled form of programmed cell death (PCD). In 2018, necroptosis was formally recognized and defined by the Cell Death Nomenclature Committee (Galluzzi et al., 2018). Morphologically, necroptosis is characterized by organelle swelling, mitochondrial dysfunction, and eventual plasma membrane rupture, distinguishing it from apoptosis (Yan et al., 2022).

At the molecular level, necroptosis is primarily governed by the receptor-interacting protein kinase (RIPK) signaling cascade. Under physiological conditions, caspase-8 functions as a key component of complex II to suppress necroptosis and promote apoptosis. However, when caspase-8 activity is inhibited, RIPK1 interacts with RIPK3 to form the necrosome complex, leading to RIPK3 activation (Bertheloot et al., 2021). Activated RIPK3 subsequently phosphorylates mixed lineage kinase domain-like protein (MLKL), triggering MLKL oligomerization and translocation to the plasma membrane. This process disrupts membrane integrity, induces ion influx and cell swelling, and ultimately results in membrane rupture and necroptotic cell death (Chen X. et al., 2014; Dovey et al., 2018; Cai et al., 2014).

**FIGURE 5:**
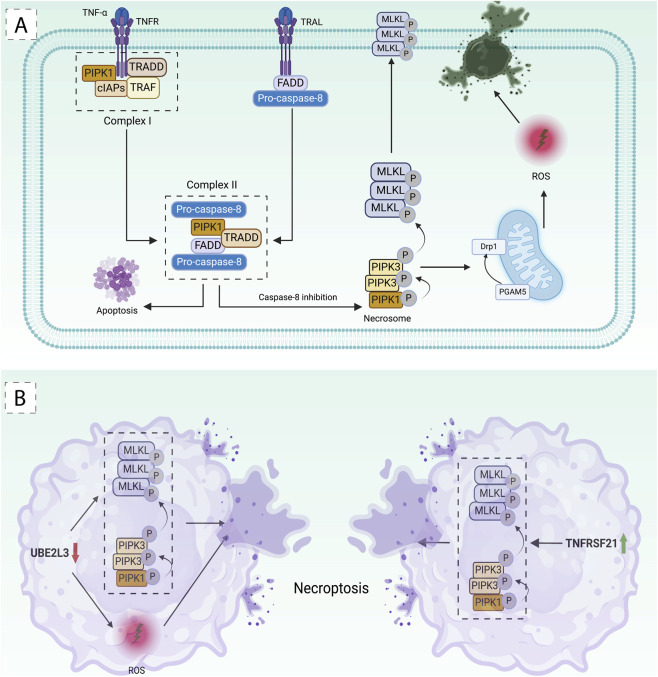
**(A)** Molecular mechanisms of necroptosis. When death receptors are activated by their ligands, complex I is formed. Through modifications such as deubiquitination, complex I can be transformed into different types of Complex II, which determine the cell’s fate (apoptosis or necroptosis). During necroptosis, the RIPK1-RIPK3 necrosome complex is formed. Activated RIPK3 phosphorylates MLKL, leading to its oligomerization and translocation to the cell membrane. This process causes membrane permeabilization, ultimately resulting in necroptosis. **(B)** Necroptosis in osteosarcoma. Knockdown of UBE2L3 or overexpression of TNFRSF21 increases the phosphorylation levels of RIP1, RIP3, and MLKL, promoting necroptosis.

In addition to its effects on membrane permeability, RIPK3-dependent necroptotic signaling has been shown to influence mitochondrial function and reactive oxygen species (ROS) production, further amplifying cellular damage and accelerating necrotic death (Wang et al., 2012; Yang et al., 2018). These findings highlight necroptosis as a tightly regulated process integrating kinase signaling, membrane disruption, and metabolic dysfunction (Figure 5A).

Although most mechanistic studies have been conducted in model systems or non-bone tumors, accumulating evidences suggest that necroptosis may also play a pivotal role in osteosarcoma. In the following section, we discuss current conclusions on necroptosis-related signaling events in osteosarcoma, as well as their implications for tumor progression and potential therapeutic targeting.

### 6.2 The role of necroptosis in osteosarcoma

Although research on necroptosis in osteosarcoma remains limited, this pro-death pathway, like other forms of PCD, is critical for osteosarcoma progression. The phosphorylation levels of RIP1, RIP3, and MLKL are key elements in necroptotic cell death. Studies have shown that the ubiquitin-conjugating enzyme E2 L3 (UBE2L3) is upregulated in osteosarcoma cells, and its knockdown promotes necroptosis by increasing the phosphorylation levels of RIP1, RIP3, and MLKL, as well as enhancing oxidative stress, thereby slowing osteosarcoma progression (Zhao et al., 2024). Additionally, TNFRSF21 also promotes necroptosis by increasing the phosphorylation levels of RIP1, RIP3 and MLKL. However, unlike UBE2L3, the action mechanism of TNFRSF21 is achieved through “overexpression” rather than “silencing” (Li X. et al., 2024). (Figure 5B).

Together, these findings highlight RIP1, RIP3, and MLKL as key mediators of osteosarcoma progression and potential therapeutic targets.

### 6.3 Natural products bidirectionally regulate necroptosis and ultimately promote osteosarcoma cell death

During tissue homeostasis and function maintaining, the proliferation and death of normal cells must be precisely balanced. However, once this balance is disrupted, cells may undergo abnormal proliferation or evade PCD, leading to benign tumors or the transformation of normal cells into malignant tumors. Steroidal saponins, including Spicatoside A (SpiA) from *L. platyphylla* roots, have demonstrated potential antitumor effects in osteosarcoma treatment. SpiA downregulates the phosphorylation of RIP, RIP3, and MLKL, successfully inhibiting necroptosis and preventing the activation of this cell death pathway. Fortunately, although spiA inhibits necroptosis, it still induces autophagy and apoptosis by enhancing ROS generation and suppressing AKT signaling pathway. Ultimately, this dual mechanism significantly reduces the migration and invasion abilities of MG-63 osteosarcoma cells and suppresses the formation of their malignant phenotype by promoting cell death over proliferation (Yun et al., 2024b).

Additionally, the same team has investigated the anticancer effects of Vitexicarpin (Vitex) in osteosarcoma. Vitex, a flavonoid extracted from *A. apiacea*, exhibits cytotoxic, anti-inflammatory, and antitumor properties. The study has identified that Vitex shares a similar action mechanism with SpiA, as it also inhibits necroptosis by downregulating the phosphorylation levels of RIP1, RIP3, and MLKL. Furthermore, Vitex promotes autophagy and apoptosis through the AKT-PRAS40 pathway, reducing the migration and invasion abilities of osteosarcoma and suppressing their malignant phenotype (Yun et al., 2024c).

Moreover, Shikonin has been found to induce necroptosis by upregulating the expression levels of RIP1 and RIP3. Notably, the study has also demonstrated that Shikonin not only triggers necroptosis at the cellular level but also significantly prolongs the survival of mice with metastatic osteosarcoma. This evidence highlights the potential of Shikonin in treating metastatic tumors (Fu et al., 2013). This contradicts the findings of the aforementioned research team. Natural products may regulate necroptosis in osteosarcoma in both inhibitory and promoting ways, which aligns with previous studies (Martens et al., 2021). Necroptosis itself acts as a double-edged sword in cancer. As an alternative form of programmed cell death, the discovery of necroptosis has provided innovative therapeutic strategies to overcome tumor cell resistance to apoptosis (Su et al., 2016). On the other hand, as an inflammatory/immunogenic cell death mode, necroptosis may promote cancer growth and metastasis or enhance anti-tumor immune responses (Aaes et al., 2016; Zhu F. et al., 2019). It is encouraging that although natural products may influence necroptosis through various mechanisms, they typically promote tumor cell death by enhancing other cell death pathways, such as autophagy or apoptosis (Supplementary Table S5).

Necroptosis is generally regarded as a compensatory form of programmed cell death when apoptotic signaling is impaired; however, its functional positioning in osteosarcoma therapy remains poorly defined. Emerging evidence suggests that, across different osteosarcoma models, the expression and/or activation status of key necroptotic mediators such as RIPK3 and MLKL is highly context-dependent, which may partially constrain the stable activation of the necroptotic pathway. Moreover, given the intrinsic inflammatory and immunogenic properties of necroptotic cell death, its impact on tumor progression may be bidirectional, reflecting a balance between direct tumor cell elimination and inflammation-driven tumor-promoting effects. Therefore, further mechanistic investigations are required to evaluate the feasibility and safety of selectively targeting necroptosis in osteosarcoma treatment.

## 7 Comparative analysis of recurrent natural products targeting distinct PCD modalities in osteosarcoma

Several recurrent natural products, such as oridonin, triptolide, shikonin, and baicalin, demonstrate the capacity to modulate multiple PCD pathways in osteosarcoma, with differences observed in cytotoxic potency, pathway preference, and experimental models (Supplementary Table S6). Notably, oridonin has been reported to induce both mitochondria-dependent apoptosis and ferroptosis (Zhang et al., 2024; Du et al., 2019; Lu et al., 2018; Liu et al., 2014; Jin et al., 2007), while shikonin can trigger apoptosis, ferroptosis, and necroptosis depending on cellular context and dosage (Huang R. et al., 2024; Lu et al., 2024; Fu et al., 2013), and triptolide preferentially activates apoptotic and autophagic pathways through distinct signaling axes (Li et al., 2018; Kwon et al., 2013; Qin et al., 2018). Similarly, baicalin has been reported to modulate multiple programmed cell death modalities, including autophagy and ferroptosis, through distinct but potentially overlapping signaling pathways in osteosarcoma models (Pang et al., 2022; Wen et al., 2023). These effects are mediated by regulation of key molecular nodes, including caspases, Bcl-2 family proteins, RIP1/RIP3, and the SLC7A11-GPX4 axis, highlighting pronounced context-dependent PCD plasticity. Overall, the ability of these natural products to engage multiple PCD pathways highlights their potential value as sensitizers or combination agents in osteosarcoma therapy, particularly for overcoming apoptosis resistance and improving treatment outcomes.

## 8 Discussion

The dynamic balance between cell survival and death is fundamental for maintaining organismal homeostasis and normal development. Disruption of this balance underlies malignant tumorigenesis, leading to uncontrolled proliferation and evasion of PCD.

This review summarizes five types of PCD (including apoptosis, autophagy, ferroptosis, pyroptosis, and necroptosis) and their mechanisms in osteosarcoma, focusing on how natural products modulate these pathways to influence cell fate. Among these pathways, apoptosis remains the most extensively studied, primarily mediated by the mitochondrial intrinsic pathway and caspase cascade activation, and serves as a key mechanism through which many natural products induce osteosarcoma cell death. Autophagy regulation by natural compounds mainly involves LC3 conversion, upregulation of Beclin-1 and Atg proteins, and inhibition of the PI3K/Akt/mTOR pathway. Ferroptosis is centered on the antioxidant defense axis involving GPX4 and SLC7A11, while pyroptosis relies on caspase-3-mediated GSDME cleavage. Necroptosis, in contrast, is controlled by the RIPK1-RIPK3-MLKL signaling axis. Collectively, these PCD pathways represent promising targets for natural product-based therapeutic strategies in osteosarcoma. Among the various PCD modalities, apoptosis remains the most extensively characterized form of cell death in osteosarcoma; however, accumulating evidence indicates that apoptotic signaling is frequently impaired in chemoresistant tumors (He et al., 2024; Hu et al., 2022; Su et al., 2016). In this context, non-apoptotic PCD modalities such as ferroptosis and necroptosis, which operate independently of the classical caspase cascade, may provide complementary or alternative therapeutic opportunities, particularly in advanced or metastatic disease.

Although PCD offers significant potential for osteosarcoma therapy, its mechanisms are complex and highly interconnected. This complexity has led to the concept of PANoptosis, a convergent form of cell death encompassing pyroptosis, apoptosis, and necroptosis. PANoptosis is triggered by specific stimuli and displays molecular features of pyroptosis, apoptosis, and necroptosis that cannot be fully explained by any single PCD pathway (Sun et al., 2024). The multi-protein PANoptosome complex integrates key effectors from apoptosis, pyroptosis, and necroptosis, generating synergistic cytotoxicity beyond individual PCD pathways (Samir et al., 2020).

Recent studies in osteosarcoma have shown that exosomes from bone marrow mesenchymal stem cells (BMSCs) carrying fibroblast growth factor-inducible 14 (FN14) activate the NF-κB signaling pathway. This activation induces PANoptosis in osteosarcoma cells, suppressing tumor growth and enhancing survival in murine models (Wang L. et al., 2025). The selective activation of PCD pathways in distinct microenvironments allows osteosarcoma cells to adaptively evade therapeutic stress. Although PCD regulation is critical for osteosarcoma therapy, targeting a single PCD pathway is often insufficient. The intricate crosstalk among PCD pathways creates a “back door” for tumor cells, increasing the likelihood of chemoresistance when therapies target a single cell death pathway. Therefore, elucidating the interactions among different PCD types in osteosarcoma is essential for precisely targeting key nodes and developing effective combination therapies.

Fortunately, current research shows that some natural products exhibit multi-regulatory effects. Nevertheless, it should be noted that evidence supporting PANoptosis in osteosarcoma is still limited and largely derived from a small number of experimental studies. The molecular composition, upstream triggers, and spatiotemporal regulation of PANoptotic signaling in osteosarcoma remain poorly defined. Moreover, whether PANoptosis represents a dominant tumor-suppressive mechanism or a context-dependent adaptive response influenced by the tumor microenvironment requires further clarification. At present, PANoptosis should be regarded as a promising but exploratory concept in osteosarcoma research rather than a fully established therapeutic target.

An increasing body of evidence indicates that the regulatory mechanisms of PCD in osteosarcoma cannot be fully understood without considering the TME (Zhou Y. et al., 2020; Liu Y. et al., 2024). However, current studies investigating natural product-induced PCD in osteosarcoma are largely confined to simplified *in vitro* tumor cell models, with limited consideration of key microenvironmental factors such as immune cell infiltration, hypoxia, and metabolic stress. These TME components not only shape osteosarcoma progression but also critically influence the functional outcomes of distinct PCD modalities.

Some natural products not only directly induce PCD in osteosarcoma cells but also exert indirect anti-tumor effects by modulating bone-associated cells within the tumor microenvironment. For example, Triptolide significantly inhibits osteoclast formation by suppressing the NF-κB signaling pathway (Huang et al., 2015), while Parthenolide (PTL) exerts anti-resorptive effects by interfering with ROS pathways (Zawawi et al., 2015). Given that osteoclasts are key regulatory cells in the osteosarcoma microenvironment, whose hyperactivity leads to osteolytic lesions and facilitates tumor cell invasion, migration, and distant metastasis, these natural compounds may indirectly inhibit tumor progression by suppressing osteoclast function and restoring bone metabolic balance, thereby reshaping the local microenvironment.

Notably, some natural products may exhibit selective effects between tumor cells and normal bone cells. Tumor cells, with their higher metabolic activity and greater capacity for uptake of exogenous substances, tend to be more sensitive to these agents (Efferth et al., 2020). One study identified that osteoblasts maintained over 80% viability at all tested concentrations of Curcumin, while the viability of MG-63 osteosarcoma cells dropped to about 50% at 10 μM, suggesting a stronger inhibitory effect of Curcumin on tumor cells (Chang R. et al., 2014). This “metabolic selectivity” has also been validated in hematologic malignancies. For instance, PTL preferentially targets acute myeloid leukemia stem and progenitor cells in NOD/SCID mice and demonstrates superior efficacy compared to conventional chemotherapy agent cytarabine (Guzman et al., 2007). Additionally, PTL has been shown to induce significant apoptosis in chronic myeloid leukemia cells with minimal impact on normal hematopoietic cells (Flores-Lopez et al., 2018). Different PCD pathways in osteosarcoma interact with one another and are regulated by several key signaling networks, including PI3K/AKT/mTOR, MAPK, ROS, and p53. These pathways do not function as simple linear cascades but form a highly interconnected network, in which shared nodes can be differentially activated depending on cell type, metabolic status, and microenvironmental cues. In this context, natural products, owing to their inherent multi-target properties, are capable of simultaneously modulating multiple signaling nodes, cell death pathways, and microenvironmental components. Such pleiotropic activity is not a limitation; rather, it may be particularly well suited to the complex heterogeneity of osteosarcoma, providing a theoretical basis for the development of combination therapies and systems-oriented natural product-based treatment strategies.

Despite the encouraging preclinical evidences, natural products with true clinical translational potential remain relatively limited. A search of the ClinicalTrials.gov database (https://clinicaltrials.gov/) has identified that several natural compounds are included in clinical trials for various cancers. For instance, Curcumin (NCT02321293, NCT03192059), Capsaicin (NCT00003610), Berberine (NCT03281096), and AG (NCT01993472) are currently under evaluation in different cancer types. In osteosarcoma-related clinical trials, Curcumin (NCT00689195) and the natural product-derived chemotherapeutic agent Paclitaxel (NCT02945800) have also been studied, highlighting the potential clinical applications of natural compounds in osteosarcoma treatment.

## 9 Research shortcomings and prospects

Despite the initial progress in studies on natural product-mediated regulation of PCD for osteosarcoma treatment, several limitations remain. On the one hand, nearly all current investigations are confined to *in vitro* or animal models, with limited validation in human subjects. On the other hand, most experiments have focused primarily on traditional osteoblastic-type osteosarcoma models. To date, the majority of studies have centered on conventional osteoblastic osteosarcoma cell lines such as MG-63, U-2 OS, and 143B. These cell lines exhibit varying degrees of invasiveness and tumorigenicity, for instance, 143B and U-2 OS cells have high metastatic and migratory potential, particularly to the lungs, while MG-63 cells display strong clonogenic capacity (Lauvrak et al., 2013). The specific experimental conditions, including the cell lines, compound concentrations, and observed effects, are summarized in Supplementary Tables S1-S5, providing a clear overview of the models used and supporting the interpretation of their biological relevance. However, there is a lack of systematic validation of natural product-mediated PCD regulation in other histologically distinct subtypes of osteosarcoma. This gap limits the generalizability and translational potential of current findings. In addition, natural products themselves pose challenges such as complex extraction processes, limited yield, poor stability, and low bioavailability, all of which hinder their clinical application and large-scale development. In recent years, several strategies have been explored to address these limitations. For example, nanoparticle-based delivery systems, liposomal encapsulation, and polymeric nanocarriers have been developed to improve the solubility, stability, and tumor-targeting capacity of natural compounds. In addition, structural modification and prodrug design have also been applied to enhance pharmacokinetic properties and therapeutic efficacy, providing promising opportunities for improving the translational potential of natural products in osteosarcoma therapy.

Beyond the five well-characterized PCD modalities, namely, apoptosis, autophagy, ferroptosis, pyroptosis, and necroptosis, emerging forms such as NETotic cell death, lysosome-dependent cell death, copper-induced cell death, and sodium overload-induced necrotic cell death have been reported. Their functional roles in osteosarcoma remain largely unexplored. The effects of natural products on PCD within the tumor microenvironment, including immune cell infiltration, hypoxia, metabolic stress, and crosstalk among different PCD pathways, are also insufficiently characterized. These knowledge gaps highlight the need for comprehensive mechanistic studies that integrate both cellular and microenvironmental contexts.

Addressing these limitations will require several approaches. The development of nanoparticle-based delivery systems and structural modifications of natural products may improve pharmacokinetics, stability, and tissue specificity. Combination therapeutic strategies, together with high-throughput screening and CRISPR-based genetic technologies, can facilitate the systematic identification of novel PCD-related targets and enhance treatment efficacy. Subtype-specific osteosarcoma models should be leveraged to evaluate differential sensitivities across histological subtypes, and clinically feasible PCD targets should be prioritized. Ultimately, the efficacy, safety, and translational potential of candidate compounds need rigorous validation through preclinical studies and prospective clinical trials. Integrating multidimensional mechanistic studies with advanced delivery strategies and molecular profiling-guided approaches will help establish natural products as effective, safe, and precise therapeutic options for osteosarcoma, providing a robust foundation for individualized treatment strategies and their potential incorporation into precision medicine.

## 10 Conclusion

PCD plays a key role in the genesis, development and treatment of osteosarcoma. Tumor cells achieve a survival advantage by escaping PCD mechanisms, which becomes a major challenge to clinical treatment. Natural products exhibit unique potential in modulating PCD. Although natural products present a promising direction for osteosarcoma therapy, current evidences are largely preclinical. Future high-quality clinical trials are warranted to rigorously evaluate their therapeutic potential, efficacy, and safety. Despite the existing challenges, the potential of utilizing natural products to treat osteosarcoma through modulating PCD remains highly promising and merits further investigation.

## Glossary

ACSL4: Acyl-CoA synthetase long-chain family 4
AHSA1: ATPase homolog 1-like protein
AMPK: 5′AMP-activated protein kinase
Apaf-1: apoptotic protease-activating factor 1
ASC: apoptosis-associated speck-like protein
Bak: Bcl-2 associated K protein
Bax: Bcl-2 associated X protein
Bcl-2: B-cell lymphoma-2
Bid: BH3-Interacting domain death agonist
BMSCs: bone marrow mesenchymal stem cells
CHOP: C/EBP homologous protein
circRNAs: circular RNAs
CLIC4: chloride intracellular channel protein 4
CYC1: cytochrome C1
Cyto-C: Cytochrome c
ΔΨm: mitochondrial membrane potential
ER: Endoplasmic reticulum
EZH2: enhancer of zeste homolog 2
FADD: Fas-associated death domain
FBP1: FUSE-binding protein 1
FER1L4: Fer-1-like protein 4
FLIP: Fas-associated death domain-like protein
FSP1: Ferroptosis suppressor protein 1
GNA: gambogenic acid
GPX4: Glutathione peroxidase 4
HDAC: histone deacetylase
HSP90: Heat shock protein 90
IRE1α: inositol requiring protein-1α
JNK: c-Jun N-terminal kinase
KLF4: Kruppel-like factor 4
lncRNAs: long-chain non-coding RNAs
LPCAT3: lyso-phosphatidylcholine acyltransferase-3
MAPK: Mitogen-activated protein kinase
MAT2A: methionine adenosyltransferase 2 alpha
M-CSF: Macrophage colony-stimulating factor 1
miRNAs: microRNAs
MLKL: Mixed Lineage Kinase domain-like
m6A: N6-methyladenosine
mTOR: mammalian target of rapamycin
ncRNAs: non-coding RNAs
NF-κB: Nuclear factor kappa-B
Panx1: Pannexin 1
PCD: Programmed cell death
PERK: protein kinase RNA-like endoplasmic reticulumkinase
PI3K-III: Class III phosphatidylinositol 3-kinase
PI3P: Phosphatidylinositol 3-phosphate
PKMYT1: protein kinase membrane-associated tyrosine/threonine 1
PRRs: pattern recognition receptors
PUFAs: polyunsaturated fatty acids
RBM15: RNA Binding Motif Protein 15
RIPK: receptor-interacting protein kinase
SH2: Src homology 2
**SystemXc** ^**-**^: Cystine-glutamate antiporter
TBRG4: Transforming growth factor beta regulator 4
TME: tumor microenvironment
TNF-α: Tumor necrosis factor alpha
TRADD: TNF receptor-associated death domain protein
TRAF2: TNF receptor associated factor 2
TRAIL: tumor necrosis factor-related apoptosis-inducing ligand
TRPV1: transient receptor potential vanilloid 1
TSSC3: tumor suppressor STF cDNA 3
ULK1: UNC-51-like kinase
UPR: Unfolded protein response
VEGF: Vascular endothelial growth factor
