# Supplementary material for: Natural products reshape osteosarcoma cell fate: promoting cell death
Source: Front Pharmacol. 2026 Jun 18;17:1729552. doi: 10.3389/fphar.2026.1729552 (PMC13323253; doi:10.3389/fphar.2026.1729552)
Supplement: Supplementary file 1 [file Table1.docx]

**Table S1 Natural products promote osteosarcoma cell apoptosis**

| Natural product | | *In vivo* efficacy evaluation | | | | | | Osteosarcoma cells  used *in vitro* | IC50 (24h) | Biomarker Changes | Apoptotic pathway | Reference |
| --- | --- | --- | --- | --- | --- | --- | --- | --- | --- | --- | --- | --- |
| Name | Chemical structure | Administration | | | | Tumor xenograft model establishment | |  |  |  |  |  |
|  |  | Dosage  (mg/kg) | Frequency | Duration | Route | Injection method | Cell and number |  |  |  |  |  |
| Oridonin | 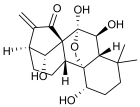 | 30 | Once every two days | 21 days | Intraperitoneal injection | Subcutaneous | HOS,  5×10^6^ | MG-63, HOS | 12.29±1.02μM (MG-63) 13.22±0.86μM (HOS) | Upregulation of Bax, Caspase-3, and Caspase-9, while inhibition of Bcl-2. Activation of PPAR-γ and suppression of Nrf2 pathway | Mitochondrial pathway | [72] |
|  |  | 100 | Once a day | 28 days | Intragastric administration | Intraperitoneal | 143B,  1×10^6^ | 143B | Not mentioned | Upregulation of Caspase-3 and inhibition of Wnt/β-catenin signaling pathway | Not mentioned | [73] |
|  |  | —— | | | | | | U-2 OS, MG-63, Saos-2 | Not mentioned | Activation of caspase-9 and caspase-3, cleavage of PARP, while downregulation of IAPs expression. Inhibition of Akt signaling pathway and activation of MAPK signaling pathway | Mitochondrial pathway | [74] |
|  |  | —— | | | | | | 143B, U-2 OS | 9.716μM (143B)  10.68μM (U-2 OS) | Upregulation of Bax and cleaved caspase-3 expression, while downregulation of Bcl-2 expression | Mitochondrial pathway | [75] |
|  |  | —— | | | | | | U-2 OS | 30μM | Upregulation of Bax, caspase-3, and caspase-9 expression, while downregulation of Bcl-2 expression | Mitochondrial pathway | [76] |
| Wogonin | 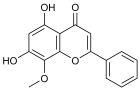 | —— | | | | | | U-2 OS | Not mentioned | Upregulation of Bad, Bax, caspase-9, caspase-3, AIF (Apoptosis-Inducing Factor), Endo G (Endonuclease G), Fas/CD95, caspase-8, GADD153 (Growth Arrest and DNA Damage-Inducible Protein 153), GRP78 (Glucose-Regulated Protein 78), ATF-6α (Activating Transcription Factor 6α), calpain 1, calpain 2, and caspase-4 expression, while downregulation of Bcl-2 expression | Mitochondrial pathway and ER stress pathway | [77] |
|  |  | —— | | | | | | CAL72 | Not mentioned | Upregulation of Bax and caspase-3, while downregulation of Bcl-2 and PARP | Mitochondrial pathway | [78] |
| Triptolide | 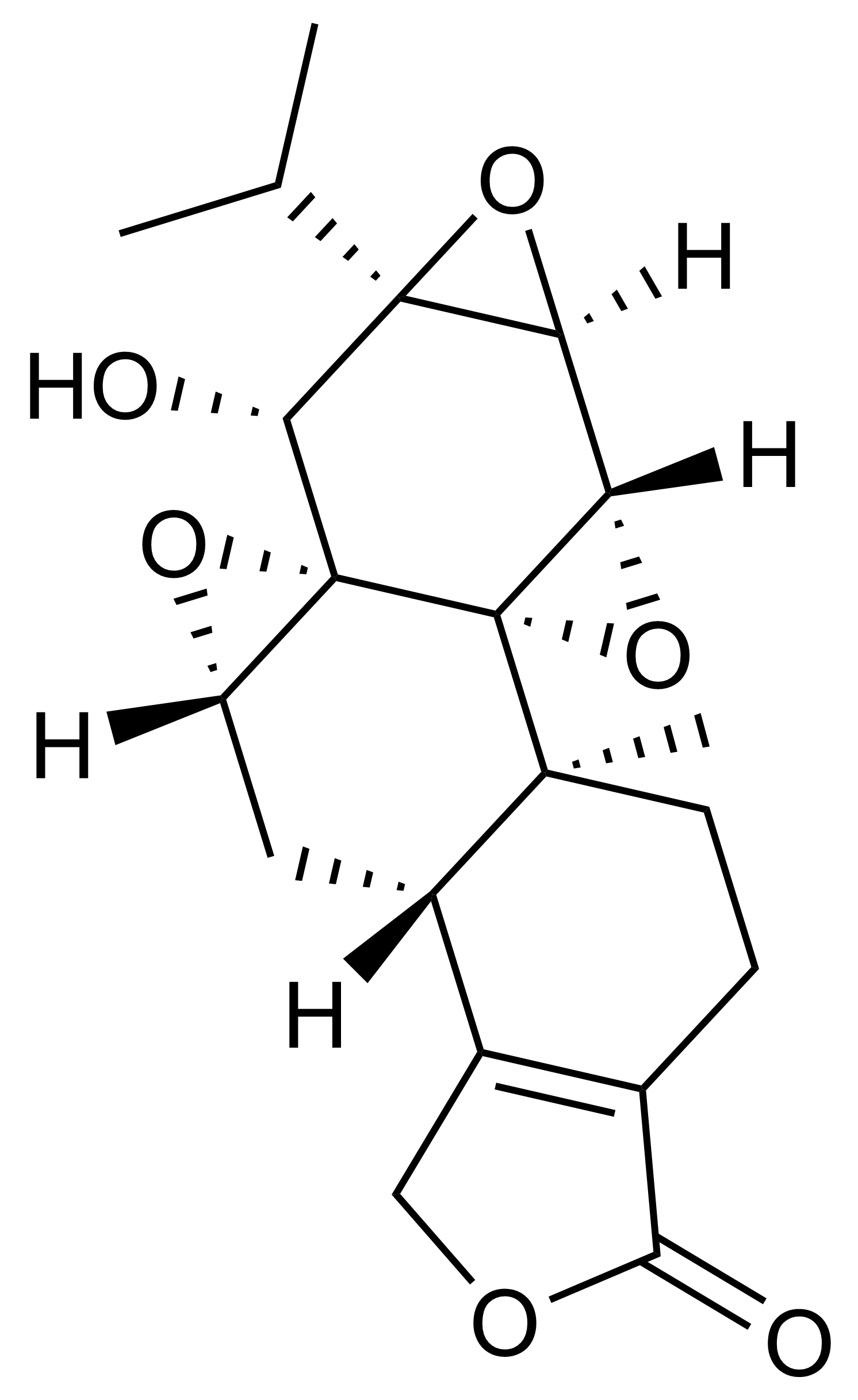 | —— | | | | | | MG-63 | Not mentioned | Upregulation of Bax and inhibition of Wnt/β-catenin signaling pathway | Not mentioned | [79] |
|  |  | 0.2 | Once a day | 10 days | Intraperitoneal injection | Intratibial | UMR-106,  2×10^4^ s | MG-63, U-2 OS，UMR-106 | 259.8nM (MG-63) 785.5nM (U-2 OS) | Activation of Bax, P53, caspase-3, and caspase-9, while downregulation of Bcl-2 and Akt, inhibits MAPK signaling pathway | Mitochondrial pathway | [80] |
|  |  | —— | | | | | | U-2 OS | 200nM | Upregulation of Fas, FasL, Bax, cytosolic cytochrome c, caspase-3, and cleaved PARP, while downregulation of procaspase-8, procaspase-9, Bcl-2, Bid, and mitochondrial cytochrome c | Mitochondrial pathway and death receptor pathway | [81] |
| Evodiamine | 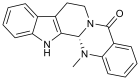 | 50 | Once a day | 28 days | Intragastric administration | Subcutaneous | 143B,  2.5×10^7^ | 143B | Not mentioned | Upregulation of caspase-3 and inhibition of the PI3K/Akt signaling pathway activate the caspase cascade | Not mentioned | [82] |
|  |  | —— | | | | | | 143B, MG-63 | Not mentioned | Upregulation of Bax, Bad, caspase-3, cleaved caspase-3, PARP, and cleaved PARP, while downregulation of Bcl-2, inhibits the Wnt/β-catenin signaling pathway. | Mitochondrial pathway | [83] |
|  |  | —— | | | | | | U-2 OS | Not mentioned | Upregulation of Bax, while downregulation of Bcl-2, caspase-3, and survivin | Mitochondrial pathway | [84] |
| Parthenolide | 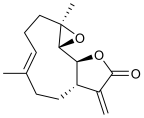 | —— | | | | | | MG-63 | Not mentioned | Inducing processes such as reactive oxygen species (ROS) generation, mitochondrial damage, nuclear translocation of AIF, and its co-localization with chromatin, activates a caspase-independent form of apoptosis | Not mentioned | [85] |
| Berberine | 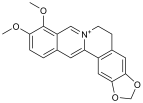 | —— | | | | | | MG-63 | 77.08μM | Upregulation of Bax and downregulation of Bcl-2. Inhibition of the MAPK signaling pathway | Mitochondrial pathway | [86] |
|  |  | 20 | Once a day | 21 days | Intragastric administration | Subcutaneous | Saos-2/MG-63, 1×10^7^ | Saos-2, MG-63 | Not mentioned | Downregulation of caspase-1 | Not mentioned | [87] |
|  |  | 50 | Once a day | Not mentioned | Intraperitoneal injection | Subcutaneous | MG-63,  1×10^7^ | MG-63, U-2 OS | Not mentioned | Upregulation of Bax and downregulation of Bcl-2. Activation of the MAPK/JNK signaling pathway | Mitochondrial pathway | [88] |
|  |  | —— | | | | | | U-2 OS | Not mentioned | Upregulation of Bax and PARP expression, along with downregulation of Bcl-2 and caspase-3. Inhibition of the PI3K/Akt signaling pathway | Mitochondrial pathway | [89] |
| Acetylshikonin | 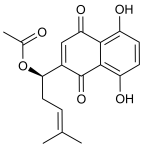 | —— | | | | | | U-2 OS | 5.6775μM | Increased cleavage of PARP, caspase-8, -9, -3, -6, and -7, upregulation of Bax, Bim, and Bad, and downregulation of Bcl-2 and Bcl-XL.Activation of the ROS/FOXO3 signaling pathway | Mitochondrial pathway | [90] |
| Shikonin | 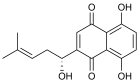 | —— | | | | | | 143B | 4.55mM | Downregulation of Bcl-2, activation of caspase-3, accompanied by PARP cleavage. Activation of the ERK signaling pathway | Mitochondrial pathway | [91] |
| Brusatol | 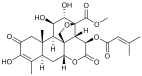 | 4 | Once a day | 14 days | Intraperitoneal injection | Subcutaneous | 143B,  1×10^6^ | 143B, U-2 OS | 51.73nm (143B)  57.73nm (U-2 OS) | Downregulation of PI3K/AKT and MAPK signaling pathway | Not mentioned | [92] |

**^[[1]](#footnote-0)^**

**Table S2 Natural products bidirectionally regulate autophagy and ultimately promote osteosarcoma cell death**

| Natural product | | *In vivo* efficacy evaluation | | | | | | | | | | Osteosarcoma cells  used *in vitro* | IC50 (24h) | Biomarker Changes | Autophagy activity （Promote/Inhibit) | Reference |
| --- | --- | --- | --- | --- | --- | --- | --- | --- | --- | --- | --- | --- | --- | --- | --- | --- |
| Name | Chemical structure | Administration | | | | | | Tumor xenograft model establishment | | | |  |  |  |  |  |
|  |  | Dosage  (mg/kg) | Frequency | | Duration | | Route | Injection method | | Cell and number | |  |  |  |  |  |
| Soy isoflavones | 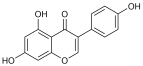 | 40 | Once a day | | 28 days | | Intragastric administration | Subcutaneous | | U-2 OS,  2×10^5^ | | U-2 OS, MG-63, Saos-2 | Not mentioned | Upregulation of LC3-II/I, PINK1, and parkin, downregulation of p62, and inhibition of the AKT/mTOR signaling pathway | Promote | [137] |
| Betulin | 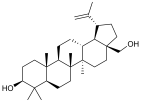 | —— | | | | | | | | | | MG-63, HOS | 14.54μM (MG-63)  11.7μM (HOS) | Upregulation of LC3-II and inhibition of the mTOR pathway | Promote | [138] |
| Butein | 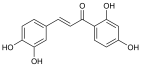 | —— | | | | | | | | | | 143B | 23.9μM | Upregulation of LC3-II and inhibition of the Akt/mTOR signaling pathway | Promote | [139] |
| Andrographolide | 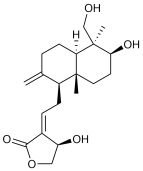 | —— | | | | | | | | | | MG-63, U-2 OS, Saos-2 | Not mentioned | Inhibition of the PI3K/Akt/mTOR signaling pathway | Promote | [140] |
| Imperatorin | 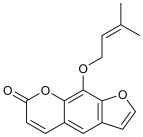 | 5 | Once every two days | | 5 days | | Intraperitoneal injection | Subcutaneous | | 143B,  1×10^6^ | | 143B, U-2 OS | 118.7μM (143B)  131.4μM (U-2 OS) | Upregulation of ULK1, Atg5, LC3-II/LC3-I, and inhibition of the PTEN-PI3K-AKT-mTOR/p21 signaling pathway | Promote | [141] |
| Cardamom | —— | 15 | Once every two days | | 20 days | | Intragastric administration | Intrafemoral | | 143B,  2 × 10^6^ | | 143B, MG-63 | 33.1μM (143B)  59.1μM (MG-63) | Inhibition of the mTOR pathway | Promote | [142] |
| Baicalin | 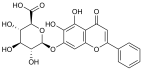 | —— | | | | | | | | | | 143B, HOS | Not mentioned | Upregulation of LC3-II and p62, inhibition of the PI3K/Akt/mTOR signaling pathway | Promote | [143] |
| Curcin C | —— | —— | | | | | | | | | | U-2 OS | 1.302μM | Upregulation of LC3-II and Beclin-1, downregulation of LC3-I, and activation of the JNK pathway | Promote | [145] |
| Peiminine | 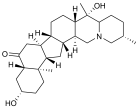 | 2 | Once every two days | | 14 days | | Intraperitoneal injection | Subcutaneous | | MG-63,  1×10^5^ | | MG-63, Saos-2 | 412μM (MG-63)  483μM（Saos-2） | Upregulation of LC3-II and Beclin-1, downregulation of p62, and activation of the ROS/JNK signaling pathway | Promote | [146] |
| Polyphyllin VI | 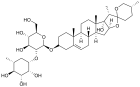 | —— | | | | | | | | | | U-2 OS | 7.33±2.85μM | Upregulation of p62, Atg-3, Atg-5, Atg-7, and LC3-II, activation of the ROS/JNK signaling pathway | Promote | [147] |
| Celastrol | 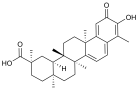 | 2 | Once a day | | 7 days | | Intraperitoneal injection | Subcutaneous | | HOS,  5×10^6^ | | HOS, MG-63 | 2.55μM (HOS)  1.97μM (MG-63) | Increase the accumulation of LC3-II and activate the ROS/JNK signaling pathway | Promote | [148] |
| Erianin | 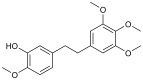 | 2 | Once a day | | 7 days | | Intraperitoneal injection | Intratibial | | 143B,  1×10^6^ | | 143B, MG-63.2 | 58.19nM (143B)  88.69nM (MG63.2) | Upregulation of LC3B-II, p62, and Beclin-1 expression, activation of the ROS/JNK signaling pathway | Promote | [149] |
| Cirsiliol | 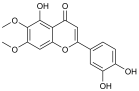 | 25 | Once every two days | | 14 days | | Intraperitoneal injection | Intratibial | | U-2 OS,  2×10^6^ | | U-2 OS | Not mentioned | Upregulation of LC3B and Beclin-1, inhibition of the AKT/FOXO1 signaling pathway | Promote | [150] |
| Dendropanoxide | —— | —— | | | | | | | | | | MG-63 | Not mentioned | Upregulation of Atg7, Beclin-1, and LC3-II, activation of the ERK1/2 signaling pathway | Promote | [151] |
| Honokiol | 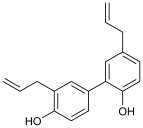 | 40 | Once a day | | 7 days | | Intraperitoneal injection | Subcutaneous | | HOS,  1×10^6^ | | HOS, U-2 OS | 17.7μM (HOS)  21.5μM (U-2 OS) | Upregulation of LC3B-II and Atg5 levels, activation of the ROS/ERK1/2 signaling pathway | Promote | [152] |
| Quercetin | 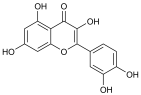 | 100 | Once a day | | 40 days | | Intragastric administration | Subcutaneous | | MG-63,  1×10^7^ | | MG-63 | Not mentioned | Upregulation of LC3B-II/LC3B-I, downregulation of p62/SQSTM1, and activation of the NUPR1 pathway | Promote | [155] |
| Triptolide | 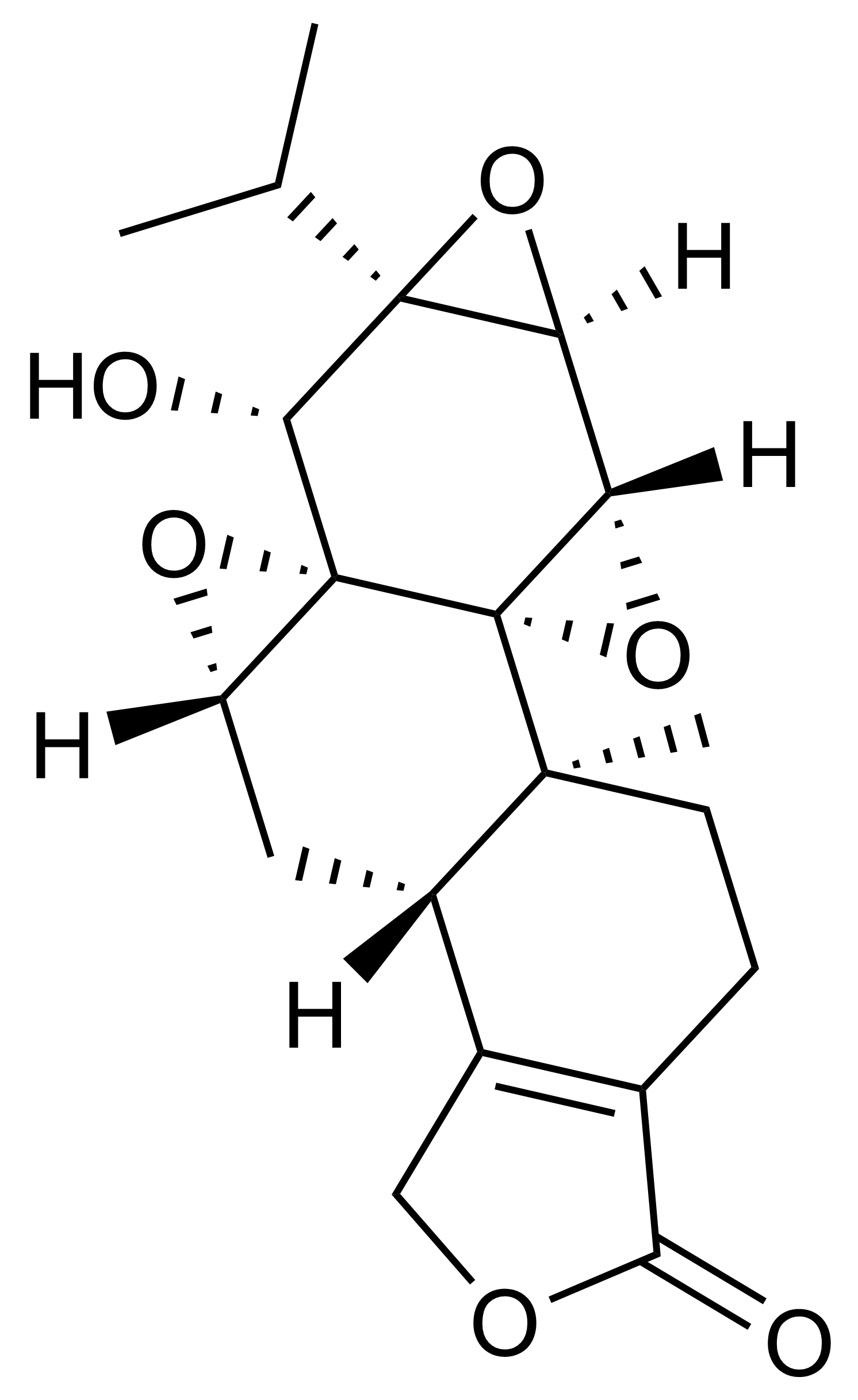 | —— | | | | | | | | | | MG-63 | Not mentioned | Upregulation of Beclin-1 expression and the LC3-II/I ratio, downregulation of p62, and inhibition of the Wnt/β-Catenin signaling pathway | Promote | [79] |
| Escin | 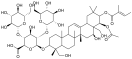 | 2.8 | Once a day | | 7 days | | Intraperitoneal injection | Intratibial | | Saos-2 OS,  5×10^6^ | | HOS, Saos-2 | 30.44μM (HOS) 29.93μM (Saos-2) | Upregulation of LC3-II, Atg5, Atg12, and Beclin-1 | Promote | [156] |
| Fraxinellone | 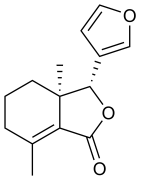 | 100 | Once a day | | 21 days | | Intragastric administration | Intratibial | | HOS,  2×10^6^ | | MG-63, HOS | 62.9μM (MG-63)  78.3μM (HOS) | Upregulation of LC3B-II/LC3B-I, Atg5, and Beclin-1 | Promote | [157] |
| Norcantharidin | 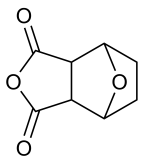 | 25 | Once every two days | | 28 days | | Intraperitoneal injection | Intraperitoneal | | HOS,  2×10^5^ | | MG-63, HOS | 128.60 ± 5.71μM（MG-63)  181.90 ± 3.41μM (U-2 OS) | Upregulation of LC3-I, downregulation of p62, and inhibition of the c-Met/Akt/mTOR signaling pathway | Promote | [158] |
| Polydatin | 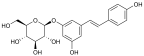 | —— | | | | | | | | | | MG-63 | Not mentioned | Inhibition of STAT3 to upregulate LC3-II, Atg12, Atg14, Beclin-1, and PI3K3 | Promote | [159] |
| Protodioscin | 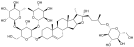 | —— | | | | | | | | | | HOS, 143B | 5.7μM (HOS)  5.1μM (143B) | Upregulation of LC3B | Promote | [160] |
| Naringenin | 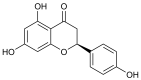 | —— | | | | | | | | | | HOS, U-2 OS | 276μM (HOS)  389μM (U-2 OS) | Upregulation of LC3-II, Atg5, and Beclin-1 | Promote | [161] |
| Licochalcone A | 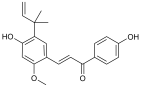 | —— | | | | | | | | | | MG-63, HOS | 31.16μM (MG-63)  29.4μM (HOS） | Upregulation of LC3A/B-II | Promote | [162] |
| Licochalcone B | 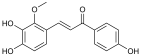 | —— | | | | | | | | | | MG-63, U-2 OS | Not mentioned | Upregulation of Atg7 and Beclin-1 | Promote | [163] |
| Anthocyanins | —— | —— | | | | | | | | | | U-2 OS | Not mentioned | Upregulation of LC3-II/LC3-I | Promote | [164] |
| Polyphyllin VII | 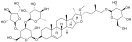 | —— | | | | | | | | | | U-2 OS | 3.48μM | Upregulated the expression of LC3-II, Atg5, Atg7, and the Atg12-Atg5 complex, and downregulated Atg12 and p62 | Promote | [165] |
| Cinobufagin | 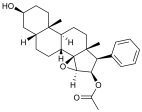 | —— | | | | | | | | | | U-2 OS | 120 mg/L | Upregulation of LC3-II/LC3-I and downregulation of p62 | Promote | [166] |
| Carnosol | 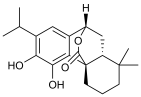 | —— | | | | | | | | | | MG-63 | 12.8 μM/ ml | Upregulation of LC3A/B-II and downregulation of p62 | Promote | [167] |
| Tanshinone IIA | 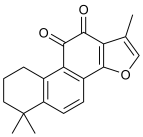 | —— | | | | | | | | | | MG-63 | Not mentioned | Upregulation of LC3-II/I and Beclin-1 | Promote | [168] |
| 11-O-Galloyl Bergenin | 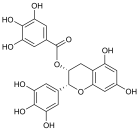 | —— | | | | | | | | | | MG-63 | Not mentioned | Upregulation of LC3-II and increased phosphorylation of p38 MAPK | Promote | [169] |
| Neohesperidin | 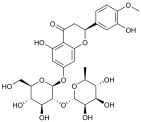 | —— | | | | | | | | | | SJSA, HOS | 29.81μ M (SJSA)  33.25μM (HOS) | Upregulation of LC3-II and Beclin-1, downregulation of p62 | Promote | [170] |
| Ginseng polysaccharide | —— | —— | | | | | | | | | | MG-63 | Not mentioned | Upregulation of LC3-II/I | Promote | [171] |
| Bufalin | 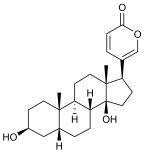 | —— | | | | | | | | | | MG-63, Saos-2 | Not mentioned | Upregulation of LC3-II/I and downregulation of p62 | Promote | [172] |
| Panax notoginseng saponins | —— | —— | | | | | | | | | | 143B | 186.2± 3.41μM | Upregulation of p53 and downregulation of LC3-II/LC3-I | Inhibit | [173] |
| Harmine | 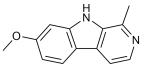 | 10 | | Once every three days | | 15 days | Intraperitoneal injection | | Subcutaneous | | MG-63,  4 × 10^5^ | MG-63, Saos-2 | 225.2μM (MG-63)  86.35μM (Saos-2) | Upregulation of LC3-II/I and phosphorylated p38 | Promote | [174] |

**Table S3 Natural products promote osteosarcoma cell ferroptosis**

| Natural product | | *In vivo* efficacy evaluation | | | | | | | | Osteosarcoma cells  used *in vitro* | IC50 (24h) | Biomarker Changes | Reference |
| --- | --- | --- | --- | --- | --- | --- | --- | --- | --- | --- | --- | --- | --- |
| Name | Chemical structure | Administration | | | | | | Tumor xenograft model establishment | |  |  |  |  |
|  |  | Dosage  (mg/kg) | | | Frequency | Duration | Route | Injection method | Cell and number |  |  |  |  |
| Capsaicin | 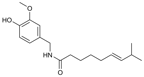 | —— | | | | | | Subcutaneous | HOS,  2×10^5^ | 143B, HOS | Not mentioned | Downregulation of GPX4 expression, increased lipid-ROS and ROS levels, and elevated intracellular Ca2+ content. Activation of PI3K/Akt and MAPK pathways | [198] |
| Casticin | 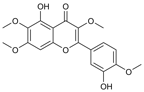 | —— | | | | | | | | 143B, MG-63 | Not mentioned | Induces Fe²⁺ overload, increases ROS levels. Upregulates the expression of HMOX1, LC3, and NCOA4, while activating the MAPK signaling pathway | [199] |
| Theaflavin-3,3′-digallate | 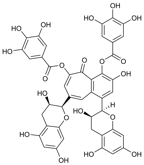 | 40 | Once a day | | | 22 days | Intragastric administration | Subcutaneous | HOS,  1×10^6^ | MG-63, HOS | 1.18μM (143B)  1.1μM (U-2 OS) | Activation of the MAPK pathway | [200] |
| Gambogenic acid | 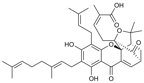 | 60 | Once a day | | | 21 days | Intragastric administration | Subcutaneous | 143B,  1×10^5^ | 143B, HOS | 1.18μM (143B)  1.1μM (U-2 OS) | Upregulation of P53, downregulation of SLC7A11 and GPX4 expression, and activation of the P53/SLC7A11/GPX4 pathway | [202] |
| Shikonin | 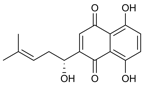 | —— | | | | | | | | 143B, MG-63 | 1.638μM (143B）  1.432μM (MG-63) | Promotes Fe²⁺ accumulation, reactive oxygen species (ROS) and lipid peroxidation formation, and malondialdehyde (MDA) production. Promotes Nrf2 ubiquitination and degradation, downregulates xCT and GPX4 | [203] |
| Shikonin | 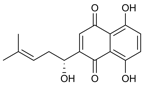 | —— | | | | | | | | MG-63, HOS | 1.279μM (MG-63) 4.055μM (HOS） | Upregulation of MDA, ROS, LPO, and Fe2+ levels, downregulation of the GSH/GSSG ratio as well as GPX4 and SLC7A11 expression. Activation of the HIF-1α/HO-1 axis | [204] |
| Baicalin | 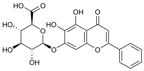 | 200 | Once a day | | | 14 days | Intraperitoneal injection | Intraperitoneal | MG-63, 2×10^5^/100μL | 143B, MG-63 | 42.32μM(MG-63)  34.2μM (HOS) | Promote Fe accumulation, ROS generation, MDA production, and downregulate the GSH/GSSG ratio. Facilitate the ubiquitination and degradation of Nrf2, and downregulate xCT and GPX4 | [205] |
| Curcumin | 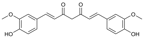 | —— | | | | | | | | MG-63, MNNG, HOS | Not mentioned | Downregulation of Nrf2 and GPX4 | [206] |
| Curculigoside | 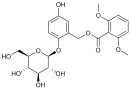 | Not mentioned | | Twice a week | | 28 days | Intraperitoneal injection | Not mentioned | SJSA-1,  5×10^7^ | 143B, HOS, U-2 OS, SJSA-1 | Not mentioned | Promote Fe accumulation and ROS generation, downregulate GPX4 | [207] |
| Bavachin | 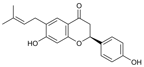 | —— | | | | | | | | MG-63, HOS | 42.32μM(MG-63）  34.2μM (HOS) | Upregulation of P53 expression, downregulation of GPX4, SLC7A11, and p-STAT3 expression | [208] |
| Oridonin | 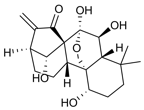 | —— | | | | | | | | 143B, U-2 OS | 9.716μM (143B)  10.68μM (U-2 OS) | Upregulation of ACSL4 expression, and downregulation of SLC7A11, GPX4, and FTH1 expression | [75] |
| Sulforaphane | 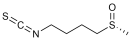 | 50 | Once every two days | | | 18 days | Intraperitoneal injection | Intratibial | 143B,  4×10^7^ | 143B, SJSA-1 | Not mentioned | Downregulation of SLC7A11 expression | [210] |

**Table S4 Natural products promote osteosarcoma cell pyroptosis**

| Natural product | | In vivo efficacy evaluation | | | | | | Osteosarcoma cells  used in vitro | IC50 (24h) | Biomarker Changes | Reference |
| --- | --- | --- | --- | --- | --- | --- | --- | --- | --- | --- | --- |
| Name | Chemical structure | Administration | | | | Tumor xenograft model establishment | |  |  |  |  |
|  |  | Dosage  (mg/kg) | Frequency | Duration | Route | Injection method | Cell and number |  |  |  |  |
| Dioscin | 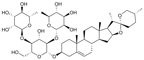 | 24 | Once every three days | 15 days | Intraperitoneal injection | Subcutaneous | MNNG/HOS, 4×106 | MNNG, HOS, MG-63, U-2 OS | 4.949μM (MNNG/HOS)  3.500μM (MG-63)  4.212μM（U-2 OS) | The activation of caspase-3 leads to the cleavage of GSDME, generating the GSDME-N fragment | [237] |

**Table S5 Natural products bidirectionally regulate necroptosis and ultimately promote osteosarcoma** **cell death**

| Natural product | | *In vivo* efficacy evaluation | | | | | | Osteosarcoma cells  used *in vitro* | IC50 (24h) | Biomarker Changes | Necroptosis  activity （Promote/Inhibit) | Reference |
| --- | --- | --- | --- | --- | --- | --- | --- | --- | --- | --- | --- | --- |
| Name | Chemical structure | Administration | | | | Tumor xenograft model establishment | |  |  |  |  |  |
|  |  | Dosage  (mg/kg) | Frequency | Duration | Route | Injection method | Cell and number |  |  |  |  |  |
| Spicatoside A | 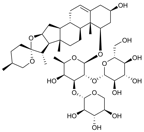 | —— | | | | | | MG-63 | Not mentioned | Downregulation of the phosphorylation of RIP, RIP3, and MLKL | Inhibit | [248] |
| Vitexicarpin | 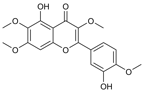 | —— | | | | | | MG-63 | Not mentioned | Downregulation of the phosphorylation of RIP, RIP3, and MLKL | Inhibit | [249] |
| Shikonin | 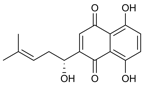 | 2 | Once every two days | 14 days | Intraperitoneal injection | Intraperitoneal | K7,  1×10^6^ | K7, U-2 OS | 2.87μM (K7)  3.18μM (U-2 OS) | Upregulation of RIP1 and RIP3 | Promote | [250] |

**Table S6 Comparison of recurrent natural products inducing distinct programmed cell death pathways in osteosarcoma**

| Natural product | | In vivo efficacy evaluation | | | | | | | | | | | Osteosarcoma cells  used in vitro | IC50 (24h) | Biomarker Changes | PCD | Reference |
| --- | --- | --- | --- | --- | --- | --- | --- | --- | --- | --- | --- | --- | --- | --- | --- | --- | --- |
| Name | Chemical structure | Administration | | | | | | | Tumor xenograft model establishment | | | |  |  |  |  |  |
|  |  | Dosage  (mg/kg) | Frequency | | Duration | | Route | | Injection method | | Cell and number | |  |  |  |  |  |
| Oridonin | 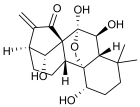 | 30 | Once every two days | | 21 days | | Intraperitoneal injection | | Subcutaneous | | HOS,  5×106 | | MG-63, HOS | 12.29±1.02μM (MG-63) 13.22±0.86μM (HOS) | Upregulation of Bax, Caspase-3, and Caspase-9, while inhibition of Bcl-2. Activation of PPAR-γ and suppression of Nrf2 pathway | Apoptosis | [72] |
|  |  | 100 | Once a day | | 28 days | | Intragastric administration | | Intraperitoneal | | 143B,  1×106 | | 143B | Not mentioned | Upregulation of Caspase-3 and inhibition of Wnt/β-catenin signaling pathway |  | [73] |
|  |  | —— | | | | | | | | | | | U-2 OS, MG-63, Saos-2 | Not mentioned | Activation of caspase-9 and caspase-3, cleavage of PARP, while downregulation of IAPs expression. Inhibition of Akt signaling pathway and activation of MAPK signaling pathway |  | [74] |
|  |  | —— | | | | | | | | | | | 143B, U-2 OS | 9.716μM (143B)  10.68μM (U-2 OS) | Upregulation of Bax and cleaved caspase-3 expression, while downregulation of Bcl-2 expression |  | [75] |
|  |  | —— | | | | | | | | | | | U-2 OS | 30μM | Upregulation of Bax, caspase-3, and caspase-9 expression, while downregulation of Bcl-2 expression |  | [76] |
|  |  | —— | | | | | | | | | | | 143B, U-2 OS | 9.716μM (143B)  10.68μM (U-2 OS) | Upregulation of ACSL4 expression, and downregulation of SLC7A11, GPX4, and FTH1 expression | Ferroptosis | [75] |
| Triptolide | 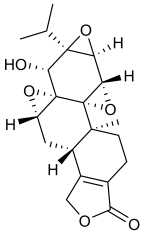 | —— | | | | | | | | | | | MG-63 | Not mentioned | Upregulation of Bax and inhibition of Wnt/β-catenin signaling pathway | Apoptosis | [79] |
|  |  | 0.2 | Once a day | | 10 days | | Intraperitoneal injection | | Intratibial | | UMR-106,  2×104 s | | MG-63, U-2 OS，UMR-106 | 259.8nM (MG-63) 785.5nM (U-2 OS) | Activation of Bax, P53, caspase-3, and caspase-9, while downregulation of Bcl-2 and Akt, inhibits MAPK signaling pathway |  | [80] |
|  |  | —— | | | | | | | | | | | U-2 OS | 200nM | Upregulation of Fas, FasL, Bax, cytosolic cytochrome c, caspase-3, and cleaved PARP, while downregulation of procaspase-8, procaspase-9, Bcl-2, Bid, and mitochondrial cytochrome c |  | [81] |
|  |  | —— | | | | | | | | | | | MG-63 | Not mentioned | Upregulation of Beclin-1 expression and the LC3-II/I ratio, downregulation of p62, and inhibition of the Wnt/β-Catenin signaling pathway | Autophagy | [79] |
| Shikonin | 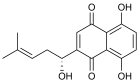 | —— | | | | | | | | | | | 143B | 4.55mM | Downregulation of Bcl-2, activation of caspase-3, accompanied by PARP cleavage. Activation of the ERK signaling pathway | Apoptosis | [91] |
|  |  | —— | | | | | | | | | | | 143B, MG-63 | 1.638μM (143B）  1.432μM (MG-63) | Promotes Fe²⁺ accumulation, reactive oxygen species (ROS) and lipid peroxidation formation, and malondialdehyde (MDA) production. Promotes Nrf2 ubiquitination and degradation, downregulates xCT and GPX4 | Ferroptosis | [203] |
|  |  | —— | | | | | | | | | | | MG-63, HOS | 1.279μM (MG-63) 4.055μM (HOS） | Upregulation of MDA, ROS, LPO, and Fe2+ levels, downregulation of the GSH/GSSG ratio as well as GPX4 and SLC7A11 expression. Activation of the HIF-1α/HO-1 axis | Ferroptosis | [204] |
|  |  | 2 | | Once every two days | | 14 days | | Intraperitoneal injection | | Intraperitoneal | | K7,  1×106 | K7, U-2 OS | 2.87μM (K7)  3.18μM (U-2 OS) | Upregulation of RIP1 and RIP3 | Necroptosis | [250] |
| Baicalin | 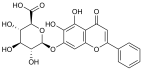 | —— | | | | | | | | | | | 143B, HOS | Not mentioned | Upregulation of LC3-II and p62, inhibition of the PI3K/Akt/mTOR signaling pathway | Autophagy | [143] |
|  |  | 200 | | Once a day | | 14 days | | Intraperitoneal injection | Intraperitoneal | | | MG-63, 2×105/100μL | 143B, MG-63 | 42.32μM(MG-63)  34.2μM (HOS) | Promote Fe accumulation, ROS generation, MDA production, and downregulate the GSH/GSSG ratio. Facilitate the ubiquitination and degradation of Nrf2, and downregulate xCT and GPX4 | Ferroptosis | [205] |

1. –: no data. [↑](#footnote-ref-0)
